# DltC acts as an interaction hub for AcpS, DltA and DltB in the teichoic acid d-alanylation pathway of *Lactiplantibacillus plantarum*

**DOI:** 10.1038/s41598-022-17434-2

**Published:** 2022-07-30

**Authors:** Nikos Nikolopoulos, Renata C. Matos, Pascal Courtin, Isabel Ayala, Houssam Akherraz, Jean-Pierre Simorre, Marie-Pierre Chapot-Chartier, François Leulier, Stéphanie Ravaud, Christophe Grangeasse

**Affiliations:** 1 Molecular Microbiology and Structural Biochemistry, CNRS UMR 5086, Université Claude Bernard Lyon 1, Lyon, France; 2 Institut de Génomique Fonctionnelle de Lyon, École Normale Supérieure de Lyon, CNRS UMR 5242, Université Claude Bernard Lyon 1, Lyon, France; 3 INRAE, AgroParisTech, Micalis Institute, Université Paris-Saclay, 78350 Jouy-en-Josas, France; 4 Institut de Biologie Structurale, CEA, CNRS UMR 5075, Université Grenoble Alpes, 3800 Grenoble, France

**Keywords:** Biochemistry, Microbiology, Structural biology

## Abstract

Teichoic acids (TA) are crucial for the homeostasis of the bacterial cell wall as well as their developmental behavior and interplay with the environment. TA can be decorated by different modifications, modulating thus their biochemical properties. One major modification consists in the esterification of TA by d-alanine, a process known as d-alanylation. TA d-alanylation is performed by the Dlt pathway, which starts in the cytoplasm and continues extracellularly after d-Ala transportation through the membrane. In this study, we combined structural biology and in vivo approaches to dissect the cytoplasmic steps of this pathway in *Lactiplantibacillus plantarum*, a bacterial species conferring health benefits to its animal host. After establishing that AcpS, DltB, DltC1 and DltA are required for the promotion of *Drosophila* juvenile growth under chronic undernutrition, we solved their crystal structure and/or used NMR and molecular modeling to study their interactions. Our work demonstrates that the suite of interactions between these proteins is ordered with a conserved surface of DltC1 docking sequentially AcpS, DltA and eventually DltB. Altogether, we conclude that DltC1 acts as an interaction hub for all the successive cytoplasmic steps of the TA d-alanylation pathway.

## Introduction

Bacterial cell wall defines cell shape and provides mechanical protection required for cell integrity. It notably encompasses a mesh-like and rigid polymer (the peptidoglycan) made of glycan strands cross-linked by peptide bonds that surrounds the whole cell^1^. In firmicutes, a major phylum of Gram-positive bacteria, the cell wall also contains secondary cell wall polymers named teichoic acids (TA). These compounds are anchored either to the peptidoglycan layer (wall teichoic acids, WTA) or to the cell membrane (lipoteichoic acids, LTA)^2–5^. WTA and LTA are generally synthesized by separated pathways and are composed of repeated units of polyol-phosphate, the best characterized structures consisting in ribitolphosphate and glycerolphosphate^6^. They both contribute to the functionality of the cell wall mediating very diverse functions ranging from cation homeostasis, autolysis, phage binding, cell division, cell wall maintenance, biofilm formation as well as adhesion to the host cells and virulence^4,7^. Being present in both pathogenic and commensal Gram-positive bacteria, TAs are often involved in host-microbe interactions, in particular they can define the immune-stimulatory potential of the bacteria^8,9^.

WTA and LTA can undergo different modifications, thus providing a structural diversity that modulates their function. Beside glycosylation and phosphocholine decoration^10^, a preponderant modification is d-alanylation which consists in the formation of an ester bond between a d-alanyl and the ribitolphosphate or the glycerolphosphate of TAs^11,12^. This modification modulates the negative charge of TAs and consequently modifies the cell surface charge and electrochemical properties. The amount of d-alanine esterified to TAs is variable and is an important mechanism by which bacteria tune teichoic acid functions^11^. Specifically, *L. plantarum* regulates the production of inflammation-related cytokines and protect the host (mice) from intestinal disorder through the d-alanine content on its cell envelope^13^. In the bacterial pathogen *Staphylococcus aureus*, the presence of d-Alanine residues on TAs changes the strain susceptibility to vancomycin and the ability to persist on human skin through evasion of cutaneous innate defenses^14,15^. d-alanylation is performed by proteins of the Dlt pathway^16–19^. This pathway begins in the cytoplasm with the acyl-carrier protein synthase AcpS that transfers a phosphopantetheine (Ppant) moiety from the coenzyme A onto the apo form of the d-alanyl carrier protein DltC^20^. Then, Ppant-DltC (holo form) interacts with the d-alanine ligase DltA to give rise to d-alanyl-Ppant-DltC^21,22^. The latter eventually interacts with the protein DltB that belongs to the membrane-bound-O-acetyltransferase (MBOAT) protein family^23^. Although this cytoplasmic pathway is supported by numerous studies, the molecular details underlying this suite of interactions remain largely unknown. While the structure of the AcpS-DltC complex has been solved at high resolution in *Bacillus subtilis*, providing a first description of the interaction between these two proteins, the contribution of Ppant has not been investigated^24^. The same is true regarding the mode of interaction between DltC and DltA that has never been clearly established. A recent study has even reported an absence of interaction between these two proteins^23^. In the same study, the mode of interaction between d-alanyl-Ppant-DltC and DltB of *Bacillus subtilis* has however been clearly determined. Concerning the extracellular step, the way d-alanine is transferred onto TAs remains highly debated. Indeed, it is proposed that DltB would either directly transfer d-alanine onto LTA, or to a lipid intermediate (phosphatidylglycerol or undecaprenyl phosphate) that would be further processed by the DltD protein for both WTA and LTA decoration^25^. Another hypothesis suggests that DltB could also directly transfer d-alanine to DltD^23^.

*Lactiplantibacillus plantarum* (former *Lactobacillus plantarum)*, a nomadic species often found in the animal oro-gastro-intestinal tract^26^, confers health benefits to its host by supporting nutrition, gut epithelial homeostasis and protection against infections^11,27–31^. This bacterium encodes for all the conserved enzymes of the TA d-alanylation pathway, including AcpS and the Dlt proteins (DltA, DltB, DltC1 and DltD). The latter are encoded by a unique gene cluster (the *pbpX2-dlt* operon). This operon also encodes for the less conserved protein DltX that consists of a single transmembrane helix. DltX was shown to be required for the decoration of TA by d-alanine in *Bacillus thuringiensis*^32^ but its role within the Dlt pathway remains largely unknown. Interestingly, a second copy of the *dltC* gene (*nc8_1214*), named here *dltC2*, is also encoded elsewhere in the genome of *L. plantarum* but its potential role in TA d-alanylation has never been investigated. Recently, evidence was provided that deletion of the *dltXABCD* genes prevents the decoration of TAs by d-alanine^33^. In addition, and most importantly, it was also demonstrated that the *dltXABCD* genes are involved in the ability of *L. plantarum* to promote *Drosophila* juvenile growth under chronic undernutrition^33^. d-alanyl decoration of TA is therefore a cardinal step for the molecular dialog underlying the mutualistic interaction between *Drosophila* and *L. plantarum*.

In this study, we first showed that individual deletion of the *acpS*, *dltA*, *dltB* genes and double deletion of *dltC1* and *dltC2* abolish TA d-alanylation as well as the beneficial interplay between *L. plantarum* and *Drosophila*. We next disentangled the suite of interactions between the cytoplasmic components of the Dlt pathway in *L. plantarum.* To this end, we solved the X-ray structures of DltC1 in complex with AcpS, and of DltA. Using NMR spectroscopy, microscale thermophoresis and molecular modeling, we characterized for the first time the molecular details of the interactions between AcpS and DltC1, DltC1 and DltA in absence or presence of Ppant, and the mode of interaction between DltC1 and DltB. Altogether, these data show that a unique surface of DltC1 is required for the sequential interaction with AcpS, DltA and finally DltB, providing thus the molecular mechanism for the cytoplasmic steps of the d-alanylation pathway.

## Results

### AcpS, DltA, DltB and DltC proteins are essential for the TA d-alanylation in *L. plantarum*

**Figure 1:**
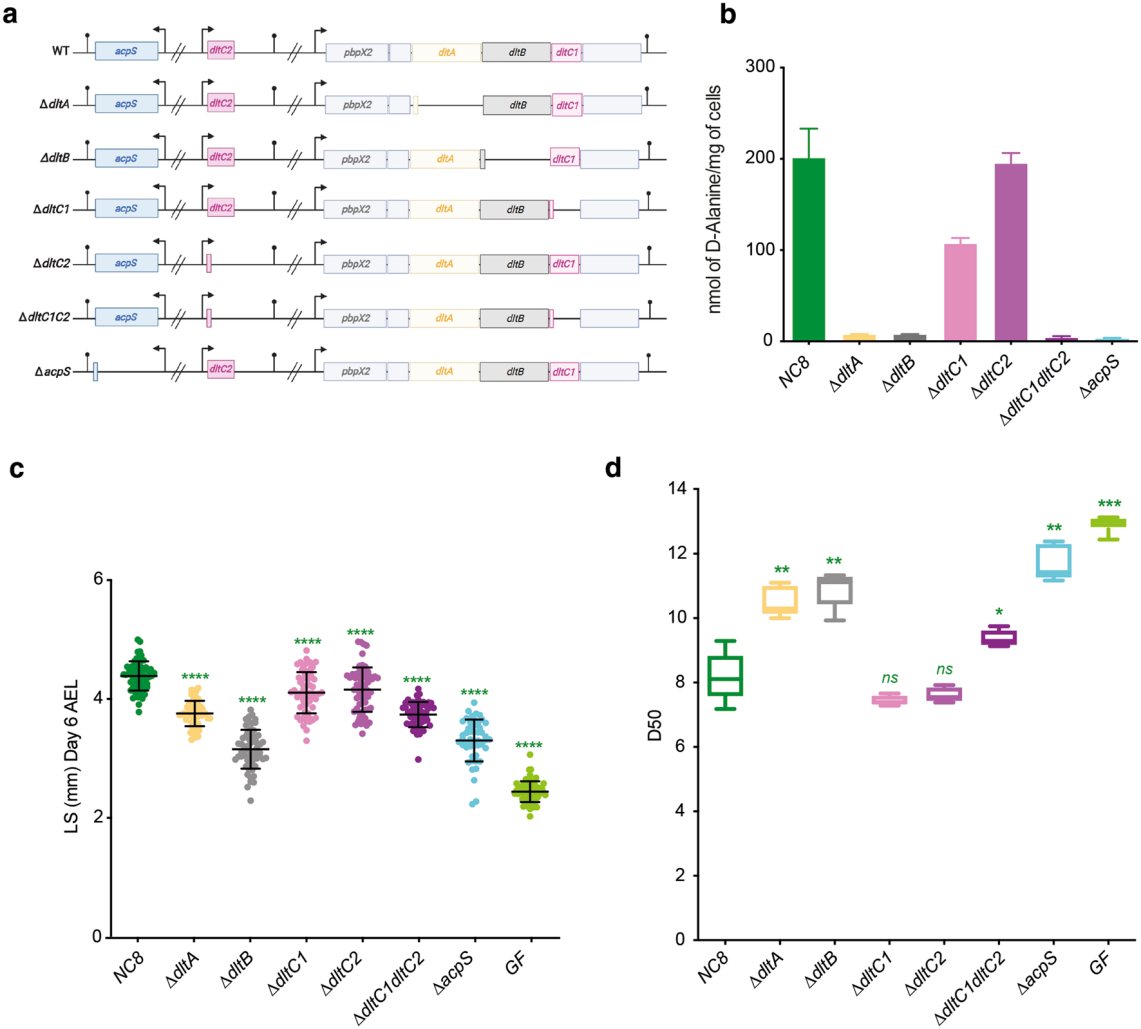
Characterization of *L. plantarum* mutants in *Drosophila*’s growth. (**a**) Genetic organization of *Lp* engineered strains: WT corresponds to *Lp*^*NC8*^ strain; ∆*dltA* corresponds to *Lp*^*NC8*^ deleted for *dltA* gene; ∆*dltB* corresponds to *Lp*^*NC8*^ deleted for *dltB* gene; ∆*dltC1* corresponds to *Lp*^*NC8*^ deleted for *dltC1* gene; ∆*dltC2* corresponds to *Lp*^*NC8*^ deleted for *dltC2* gene; ∆*dltC1C2* corresponds to *Lp*^*NC8*^ deleted for *dltC1* and *dltC2* genes; ∆*acpS* corresponds to *Lp*^*NC8*^ deleted for *acpS* gene. *in-frame* deletions were constructed through homology-based recombination with double-crossing over, in such a way that the two first triplets of the sequence are fused with the two last. (**b**) Amount of d-Ala released from whole cells of NC8 and derivative mutants by alkaline hydrolysis and quantified by HPLC. Error bars represent the standard deviations. (**c**) Larval longitudinal length after inoculation with strains *Lp*^*NC8*^, ∆*dltA,* ∆*dltB,* ∆*dltC1*, ∆*dltC2,* ∆*dltC1dltC2*, ∆*acpS* or PBS (for the GF condition). Larvae were collected 6 days after association and measured as described in the Methods section. Green asterisks illustrate statistically significant difference with *Lp*^*NC8*^ larval size; ****: *p* < 0.0001. Center values in the graph represent means and error bars represent SD. Representative graph from one out of three independent experiments. (**d**) Day when fifty percent of pupae emerge during a developmental experiment (D50) for GF eggs associated with strains *Lp*^*NC8*^, ∆*dltA,* ∆*dltB,* ∆*dltC1*, ∆*dltC2,* ∆*dltC1dltC2*, ∆*acpS* or PBS (for the GF condition). Center values in the graph represent means. Green asterisks illustrate statistically significant difference with *Lp*^*NC8*^ D50; *ns* represent absence of statistically significant difference with *Lp*^*NC8*^ D50. ***: 0.0001 < *p* < 0.001; **: 0.001 < *p* < 0.01; *: *p* < 0.05.

We first aimed at confirming that AcpS and the Dlt proteins DltA, DltC1 and DltB were individually required for the cytoplasmic steps of the TA d-alanylation pathway in *L. plantarum*. To this end, we generated the ∆*acpS*, ∆*dltA*, ∆*dltC1* and ∆*dltB* deletion mutants in the *Lp*^*NC8*^ strain by homology-based recombination and determined the amount of d-Ala esterified to TAs in each strain (Fig. 1a and b). Compared to the d-Ala level measured in the WT strain (*Lp*^*NC*8^ strain), the levels of d-Ala released from these mutants were almost undetectable, except for the ∆*dltC1* mutant in which only a decrease of 25% is measured (Fig. 1b). This confirms that AcpS, DltA and DltB are required in this pathway but suggests that a complementary DltC activity should contribute to TA d-alanylation. In addition to *dltC1* (*nc8_1734*, henceforth named *dltC1*) located within the *dlt* operon, careful inspection of the genome of *L. plantarum* identified a second gene, named *dltC2* (*nc8_1214*)*,* present in another place in the genome (Fig. 1a), which encodes a protein sharing 88.75% identity and 96.25% similarity with DltC1 (Supplementary Fig. 2a). The two proteins actually differ only in 9 residues. We therefore hypothesized that DltC2 could also contribute to TA d-alanylation. While we did not detect any decrease of TA-alanylation in *Lp*^*NC*8^ devoid of the *dltC2* gene, we observed that TA d-alanylation was completely abolished in the double deletion mutant **∆***dltC1dltC2* (Fig. 1b). Together, these results show that DltC1 is the main enzyme at play in the TA d-alanylation pathway but also suggest that both DltC1 and DltC2 could work in synergy to allow d-alanylation of TA.

The *pbpX2-dlt* operon of *L. plantarum* is required to promote the *Drosophila* juvenile growth under chronic undernutrition^33^. To analyze the respective contribution of each gene in this functional setting, we assessed the impact of each mutant strain on the ability of *L. plantarum* to support the growth of *Drosophila* larvae. To this end, we determined both the larval size at six days post-inoculation and the developmental timing of the germ free (GF) individuals and individuals mono-associated with the wild-type or ∆*acpS,* ∆*dltA,* ∆*dltB or* ∆*dltC1dltC2 Lp* strains (Fig. 1c). ∆*acpS,* ∆*dltA,* ∆*dltB or* ∆*dltC1dltC2* associated larvae were significantly smaller than those associated with the wild type *Lp*^*NC8*^ strain*.* In addition, their larval development was also severely delayed (Fig. 1d). Of note, single deletion of *dltC1* or *dltC2* induced only slight defects reinforcing the notion that *dltC* genes encode redundant and synergistic activities. These results therefore showed that AcpS, DltA, DltB and, DltC1 or DltC2, are needed for TA d-alanylation and contribute to *L. plantarum* beneficial activity towards its animal host exemplified here by the promotion of *Drosophila* juvenile growth.

### Structural characterization of the AcpS/DltC1 complex

The first step in the d-alanylation mechanism of TA is the transfer of the phosphopantetheinyl moiety of coenzyme A (Ppant) (Supplementary Fig. 2) to the DltC protein by the Phosphopantetheinyl transferase AcpS. During this process, the Ppant is covalently attached to a conserved Serine residue (Ser38) (Supplementary Fig. 1a) converting DltC1 or DtC2 from an apo-form to the active holo-form. The high sequence identity between DltC1 and DltC2 suggested that the mode of interaction with AcpS is likely conserved (Supplementary Fig. 1a). Therefore, and as the *dltC1* gene is located in the *dlt* operon, we decided to focus our structural analyses on the AcpS/DltC1 complex and to transpose our observations to DltC2 to characterize its mode of interaction with AcpS.

**Figure 2:**
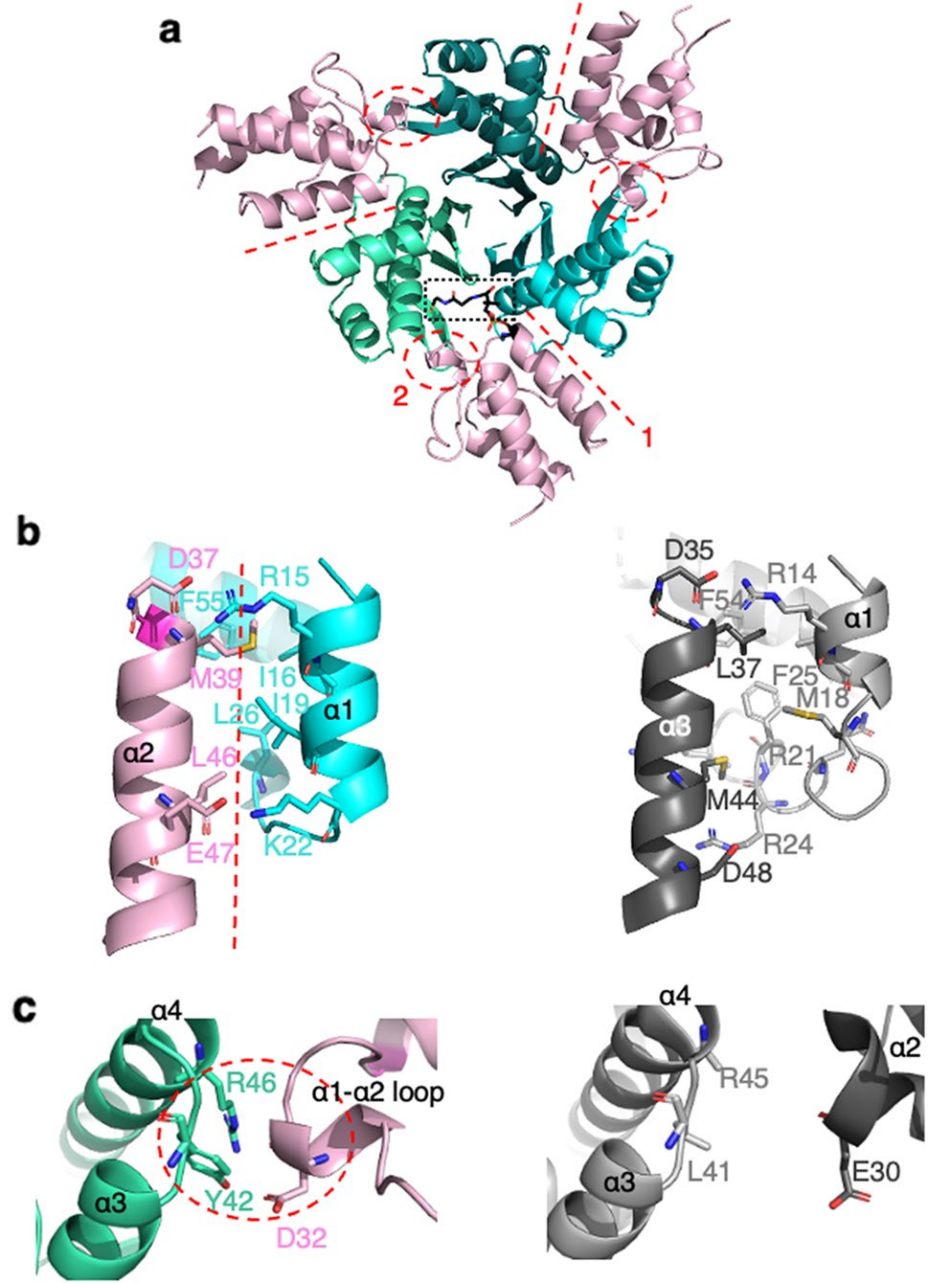
The *L. plantarum* AcpS-DltC1 complex structure. (**a**) Cartoon representation of the *L. plantarum* AcpS-DltC1 3D structure with the 3-branch AcpS star shown as a cyan-green color gradient and the DltC1 molecules in pink. The Ppant cofactor bound to one DltC1 molecule is shown as black sticks and highlighted by a black dashed-rectangle. The main contact surfaces, 1 and 2 are depicted as red dashed line and circle respectively. (**b**) Close-up on the contact surface 1. The interacting residues are shown in stick. Comparison between *L. plantarum* (left—AcpS in blue and DltC1 in pink) and *B. subtilis* (right—AcpS in light grey and DltC in dark grey—PDB entry 1F80) complex structures. (**c**) Close-up on the contact surface 2. The interacting residues are shown in stick. Comparison between *L. plantarum* (left—AcpS in green and DltC1 in pink) and *B. subtilis* (right—AcpS in light grey and DltC in dark grey).

To study the transfer of Ppant, we overexpressed and purified *L. plantarum* AcpS and DltC1 fused to a His-tag from *Escherichia coli* cells. Then, by mixing the two Ni-affinity purified proteins, we performed size exclusion chromatography to isolate the AcpS-DltC1 complex (Supplementary Fig. 3). The fractions containing the AcpS-DltC1 complex were then pooled to conduct the crystallization trials. High quality diffracting crystals were obtained and the crystal structure was solved at 1.88 Å resolution. The data collection and refinement statistics are summarized in Table 1. The AcpS-DltC1 complex clearly adopts a 3:3 stoichiometry with a 3D arrangement similar to that of the AcpS-DltC complex from *B. subtilis*^24^ with a rmsd (root mean square deviation) of 1.062 Å on 341 residues. This arrangement forms a 3-branch AcpS star that is decorated with 3 DltC1 molecules, each of them being at the interface between two AcpS subunits (Fig. 2a).

**Table 1:** Data collection and refinement statistics.

	AcpS/DltC1	DltA
Wavelength (Å)	0.9677	0.97856
Resolution range (Å)	43.79–1.88 (1.92–1.88)	47.36–2.01 (2.06–2.01)
Space group	P 21 21 21	P 1 21 1
Unit cell (Å, °)	68.54, 94.89, 98.71, 90, 90, 90	68.52, 53.57, 102.07, 90, 96.78, 90
Total reflections	709,889 (37,794)	216,494 (15,463)
Unique reflections	52,945 (3096)	49,012 (3478)
Multiplicity	13.4 (12.2)	4.4 (4.4)
Completeness (%)	99.3 (91.2)	99.4 (95.1)
Mean I/sigma (I)	9.1 (1.4)	7.2 (0.9)
Wilson B-factor	27.77	25.76
R-merge	0.223 (1.969)	0.173 (1.452)
R-meas	0.232 (2.053)	0.196 (1.650)
R-pim	0.063 (0.569)	0.0090 (0.770)
CC1/2	0.997 (0.646)	0.994 (0.522)
Reflections used in refinement	50,507 (3468)	44,283 (2692)
Reflections used for R-free	2012 (132)	1806 (119)
R-work	0.2156 (0.4717)	0.2093 (0.3135)
R-free	0.2624 (0.5017)	0.2667 (0.3968)
Number of non-hydrogen atoms	4922	6642
Macromolecules	4534	6076
Ligands	35	56
Solvent	353	510
Protein residues	590	792
RMS (bonds)	0.008	0.008
RMS (angles)	1.01	0.99
Ramachandran favored (%)	96.18	96.56
Ramachandran allowed (%)	3.30	3.05
Ramachandran outliers (%)	0.52	0.38
Rotamer outliers (%)	1.01	1.06
Clashscore	6.10	5.28
Average B-factor	34.22	31.3
Macromolecules	34.03	30.96
Solvent	36.17	33.83
Ligand	38.46	45.86

Statistics for the highest-resolution shell are reported in parentheses.

Compared to the available structures of homologous proteins^23,24,34–38^, the global fold of AcpS and DltC1 is not modified upon complex formation (Supplementary Figs. 4 and 5). Indeed, each AcpS monomer adopts an α/β fold and shows high structural similarity with already published AcpS structures with a rms difference of Cα atoms of 0.7 Å compared to AcpS structure from *B. subtilis*^24^. The longest α-helix (α4 ranging from residues 44 to 65), is wrapped on one side by a three-stranded antiparallel β-sheet (β1, β4 and β5) and a β-ribbon composed of two strands (β2 and β3) and on the other side by three small α-helices and a 3–10 helix (Fig. 2a and Supplementary Fig. 4). The β-sheet of each monomer is positioned at the center of the trimer and contributes to the trimerization via hydrophobic and a few hydrophilic interactions.

Regarding DltC1, its structure exhibits a right-handed helix bundle topology, a typical fold shared with the other DltC proteins whose structure has been solved^23,35,37^. The rms differences of the Cα atoms ranged from 1 to 1.2 Å. While the 3 main α-helices (ranging from residues 3–18, 38–52 and 69–80) are well conserved, the region connecting the α-helices are less structured in DltC1 than those in other DltC homologs. A long loop is connecting α1 and α2 while in *B. subtills* or in *L. rhamnosus* an additional 6 residues helix is observed^35,37^. Between α2 and α3 the short perpendicular helix usually described in homologous structures is replaced by a 3–10 helix between residues 58–61(Fig. 2a and Supplementary Fig. 5).

The DltC1 catalytic Ser38 is the first residue of helix α2. A clear electron density, corresponding to a phosphate moiety of Ppant, was observed covalently linked to the Ser38 side chain in only one of the 3 subunits of DltC1 of the complex (Fig. 2a). Although the electron density of the remainder of Ppant moiety was weaker, presumably due to its intrinsic flexibility, the distal end of the prosthetic group could be modeled (Supplementary Fig. 6a). While Ppant folds back on DltC at the interface between helix α2 and α3 in the AcpS-DltC complex from *B. subtilis*, its position differs in our structure (Supplementary Fig. 6b). Indeed, although covalently linked to Ser38, it points toward a neighboring AcpS molecule and ends in the vicinity of the end of β2 and the beginning of α4. In this position, the thiol group at the extremity of the Ppant is stabilized via H-bond interactions with Glu49, Asp82 and the well conserved Arg46 of AcpS (Supplementary Fig. 6a). This Ppant location is however similar to that of the CoA molecule co-crystallized with AcpS from *B. subtilis*^24^. Considering the sequence conservation between DltC1 and DltC2 (Supplementary Fig. 1a), we used the 3D DltC1 structure to generate a 3D model of DltC2 and showed that all the structural features and binding analysis described for DltC1 are conserved in DltC2 (Supplementary Fig. 1b and c).

### Binding interface of DltC1 to AcpS

The interface between two adjacent AcpS monomers serves as a docking area for DltC1 binding (Fig. 2a). Two main contact surfaces, annotated 1 and 2 on Fig. 2a, were identified between AcpS and DltC1. The first one is the largest and locates at the interface between the helix α2 and the loop connecting helices α2 and α3 of DltC1 and the helix α1 of one adjacent AcpS monomer. It is composed of hydrophobic and ionic interactions (Fig. 2b). The two main hydrophobic contacts involve Met39 and Leu46 of DltC1. Met39 binds in a pocket formed by the side chains of Ile16 and Ile 19 of AcpS helix α1 and to the side chain of Phe55 while Leu46 interacts only with Leu26 of AcpS. Two similar clusters of hydrophobic interactions were also described in the homologous complex structure described for *B. subtilis* but amino acids are not conserved^24^ (Fig. 2b). In this contact surface 1, key ionic interactions also contribute to the stabilization of the complex. One is strictly conserved in *B. subtilis* and is established between the well conserved Arg15 of AcpS and Asp37 of DltC1, the latter being located just before the reactive Serine. A second salt bridge is formed between Glu47 of DltC1 and Lys22 of AcpS (replaced by an Arg residue in *B. subtilis*) and is the only interaction observed at the terminal end of helix α2 of DltC1. The second contact surface (contact surface 2 on Fig. 2a) involves a salt bridge between DltC1 and the second neighboring AcpS molecule and further stabilizes the *L. plantarum* complex. The Asp32 of DltC1 located in the long loop between helices α1 and α2 forms a strong ionic interaction with Arg46 of AcpS and an H-bond with Tyr42. Although Asp32 from DltC1 and Arg46 from AcpS are conserved, these interactions were not described in *B. subtilis* complex, the loop region between α1 and α2 adopting different conformations in both structures (Fig. 2c).

Facing these structural features, we wondered what could trigger the dissociation of the AcpS-DltC1 complex after the Ppant has been transferred to DltC1, an event necessary prior to the transfer of d-Ala as a thiol ester to the Ppant by DltA ligase. We hypothesized that Ppant transfer to each DltC1 molecule could be key by decreasing the stability of the complex leading to the release of holo-DltC1. To test this hypothesis, we produced both the apo- and holo-forms of LpDltC1. The apo form of DltC1 was obtained by mutating the catalytic Ser in Ala. On the other hand, the production of the holo-form (full Ppanted DltC1) was achieved by co-expressing both Lp*AcpS* and *DltC1* from a pRSF-Duet1 vector in *E. coli*. The presence of the Ppant modification in DltC1 was confirmed after its purification by mass spectrometry analysis (Supplementary Fig. 7). Next, purified AcpS was mixed with either apo- or holo-DltC1 and the mixtures were subjected to size exclusion chromatography. In the case of apo-DltC1 a large elution peak containing the AcpS-DltC1 complex is obtained followed by a small peak of unbound DltC1 (Supplementary Fig. 3a and c). By contrast, mixing of AcpS with Ppant-modified DltC resulted in two large separate peaks corresponding to unbound AcpS and holo-DltC1, respectively (Supplementary Fig. 3b and c). No complex formation was therefore detected demonstrating that the AcpS-DltC1 complex can no longer form when DltC1 is fully Ppanted. Microscale thermophoresis (MST) experiments also confirmed that AcpS do not interact with holo-DltC1 (Supplementary Fig. 8).

### Structural characterization of DltA

**Figure 3:**
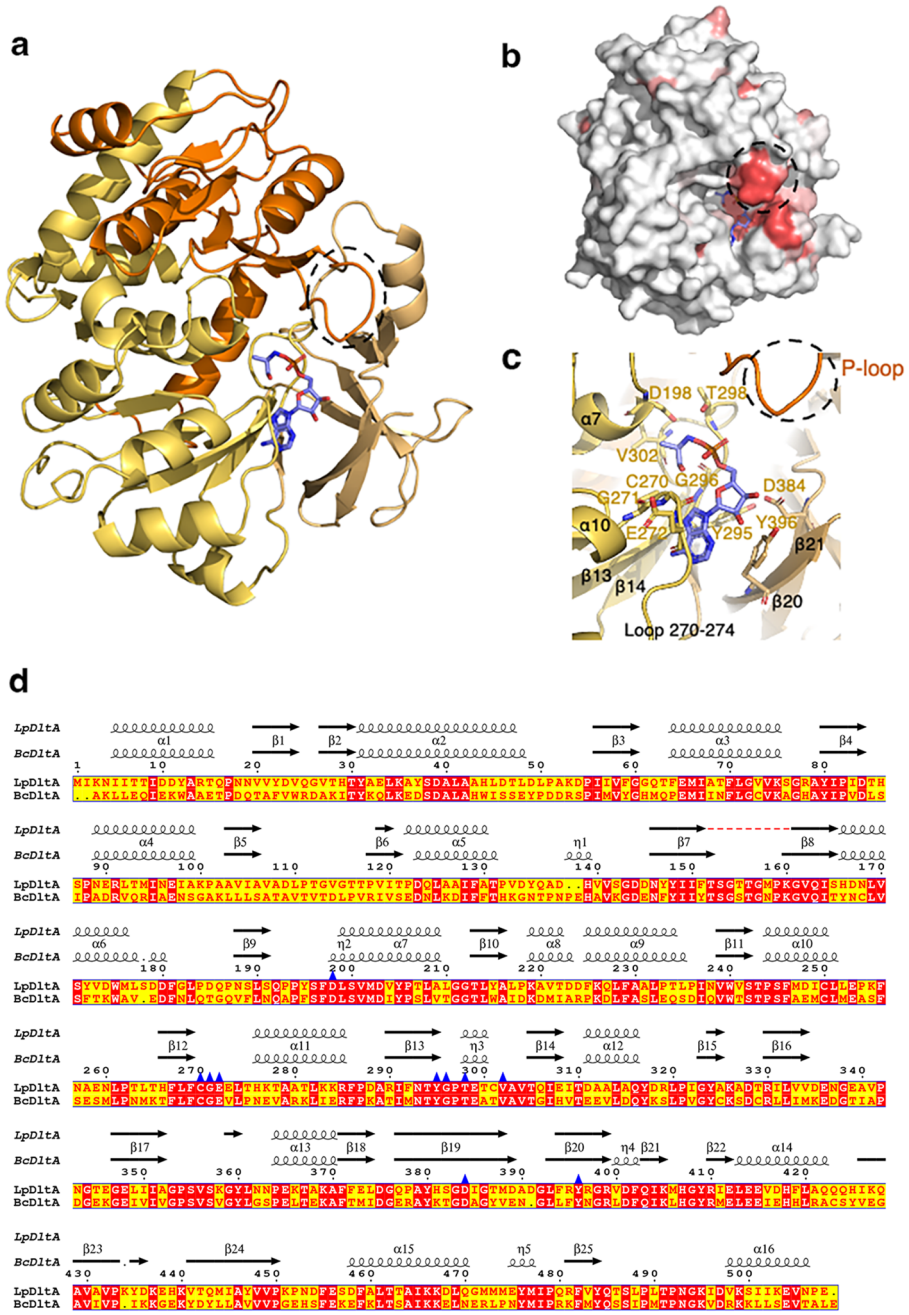
The *L. plantarum* DltA structure. (**a**) Cartoon representation of the N-terminal domain of DltA structure. The subdomains A (3–48, 188–320), B (49–187) and C (321–400) are respectively colored in orange, yellow and wheat. The d-Ala-AMP substrate is shown as blue stick. The P-loop is encircled with dashed black line. (**b**) Surface representation of the N-terminal domain of DltA colored according to sequence conservation. The color ramping from white (low score) to red (identity) locate areas of weak and strong sequence conservation. The figure was generated with ENDscript (https://endscript.ibcp.fr/) (**c**) Close-up on the substrate binding site of DltA, the d-Ala-AMP (in blue) and the residues involved in the interaction are shown as sticks (**d**) Sequence alignment between *L. plantarum* DltA (LpDltA) and DltA from *B. cereus* (BcDltA—PDB entry 3DHV). The secondary structures extracted from the X-ray structures are depicted above. The position of the LpDltA residues interacting with the d-Ala-AMP substrate are highlighted by a blue triangle. The position of the P-loop is shown with a dashed black line. The figure was generated by ESPript (https://espript.ibcp.fr/).

The d-alanine-d-alanyl carrier protein ligase (DltA) catalyzes a two-step reaction: the activation of d-Ala using ATP to form a d-alanyl-AMP intermediate and then the transfer of d-Ala as a thiol ester to the Ppant group of DltC. To investigate this mechanism, we produced and purified *L. plantarum* DltA and solved its crystal structure at 2.1 Å resolution. The data collection and refinement statistics are summarized in Table 1. The asymmetric unit of the crystal contains two DltA molecules which are largely similar (rmsd of 0.5 Å on 392 residues). Out of 508 residues of full-length DltA, the final model contains residues 3–401 in one molecule and residues 3–400 in the second molecule of the asymmetric unit. The last 100 aminoacids constituting the C-terminal minor domain are not visible in the electron density although present in the purified sample. The loop region ranging from residues 152 and 160 could only be partially modeled in one copy due to poor electron densities for residues 153 to 157 but was fully visible in the second molecule. The overall structure of the large N-terminal domain (residues 3–395) is composed of three subdomains (referred in previous studies as subdomains A, B and C^39^) two of which, A and B, sharing a similar topology with a β-sheet flanked on both sides by α -helices (Fig. 3). The last subdomain C (residues 321–400) forms a distorted β -barrel. Superimposition of the large domain of *L. plantarum* DltA with homologous DltA structures from *B. subtilis*^39^, *B. cereus*^40–42^ and *S. pyogenes* (PDB entries 3L8C, 3LGX, unpublished data) reveals high structural similarity with root mean square deviations (r.m.s.d.) in the range of 0.7–0.9 Å for 300 residues (Supplementary Fig. 9).

### Interaction between DltA and d-Ala-AMP

As crystallization was achieved in the presence of d-Ala and ATP, one molecule of d-Ala-AMP could be observed in the electron density of one DltA molecule of the asymmetric unit (Fig. 3). This position is similar to that described in the *B. cereus* DltA structure^40^ (Supplementary Fig. 9a). In the other molecule, the clear electron density allowed to position with confidence the adenine ring moiety and the d-Ala in the same position as in the other DltA molecule. However, the weaker density observed at the ribose and phosphate positions did not support the presence of the covalent bond expected between the d-Ala and the phosphate group. This suggests either a certain degree of flexibility or that we manage to trap an intermediate during the d-Ala activation by an ATP molecule. The d-Ala-AMP binding cleft of DltA is defined at the interface between loop 270–274, the β strands β13 (residues 290–295), β14 (residues 305–308) and β21 (residues 394–399) and the P-loop (phosphate binding loop or Walker A motif) comprised between residues 152 and 160 (Fig. 3b and c). More precisely, the adenine ring is sandwiched between residues Gly271 and Glu272 on one side and Tyr295 on the other side. The ribose forms three H bonds with the carboxylate group of Asp384, the side chain of Tyr396 and the hydroxyl group of Gly271. The phosphate group interacts with the side chain and the amide group of Thr298. The d-alanyl amino group appears anchored by three interactions with the carboxylate group of Asp198 and with the hydroxyl groups of Gly296 and Val302. As already described for other DltA structures mentioned above, a conserved cysteine residue 270 is positioned close to the side chain of the d-alanyl moiety participating in the d-Ala versus L-Ala specificity of the enzyme^40^ (Fig. 3c and d). Altogether, this analysis shows that this interaction web is largely similar to that of the d-Ala-adenylate described in the DltA structure from *B. cereus*^40^ with the exception that the interaction observed with the small C-terminus domain could not be confirmed due to its absence in our crystal structure. As a result, the binding cleft appears more widely opened. However, and very interestingly, our structure showed that the P-loop clearly participates to the shaping of the binding cleft whereas it was not observed in the DltA structure of *B. cereus* (see below) (Fig. 3b and c and Supplementary Fig. 9).

While most of the surface residues are poorly conserved in DltA proteins and homologous proteins forming AMP intermediates, the P-loop is highly conserved (nine residues from Thr152 to Lys 160)^43–47^ (Fig. 3b). This segment is most of the time disordered in the crystal structures but is thought to be important in the adenylation reaction. As its amino acid composition resembles that of P-loops in ATPases and GTPases^48^, it is supposed to be involved in ATP or pyrophosphate binding. In our DltA structure, the P-loop is well defined in one molecule of the asymmetric unit (Supplementary Fig. 9d). It was also defined in the ATP bound state of the *B. subtilis* DltA structure^39^ but importantly, it is the first time that it is observed in the adenylation state of a d-alanine-d-alanyl carrier protein ligase. In addition, the loop adopts here a different conformation, pointing towards the phosphate group of the substrate. It is stabilized by an H-bond between the side chain of the conserved Arg91 and the main chain of Thr152. Although no direct interaction is established with the d-Ala-AMP, a locally positively charged environment mainly through the Lys160 is created in the vicinity of the phosphate group. These new structural data suggest that the P-loop could therefore contribute to the stabilization of the substrate binding (Fig. 3b and c). Such a conformation is compatible with the position of the C-terminal domain of the *B. cereus* DltA structure obtained in complex with d-Ala-AMP^40^. However, it would clash partially with the second β-strand of the small C-terminal domain as positioned in the *B. subtilis* ATP-bound DltA structure^39^ (Supplementary Fig. 9d). It was previously suggested that movement of the C-terminal domain could occur while switching from one conformation state to the others (apo, ATP bound, adenylation and thiolation states) together with a reorganization of the P-loop conformation^39^. In light of our observations, we reconcile this apparent discrepancy as they bring structural evidence that the P-loop likely moves in concert with the C-terminus domain while DltA switches from the ATP bound to the adenylation states.

### Analysis of the DltA–DltC1 interaction

**Figure 4:**
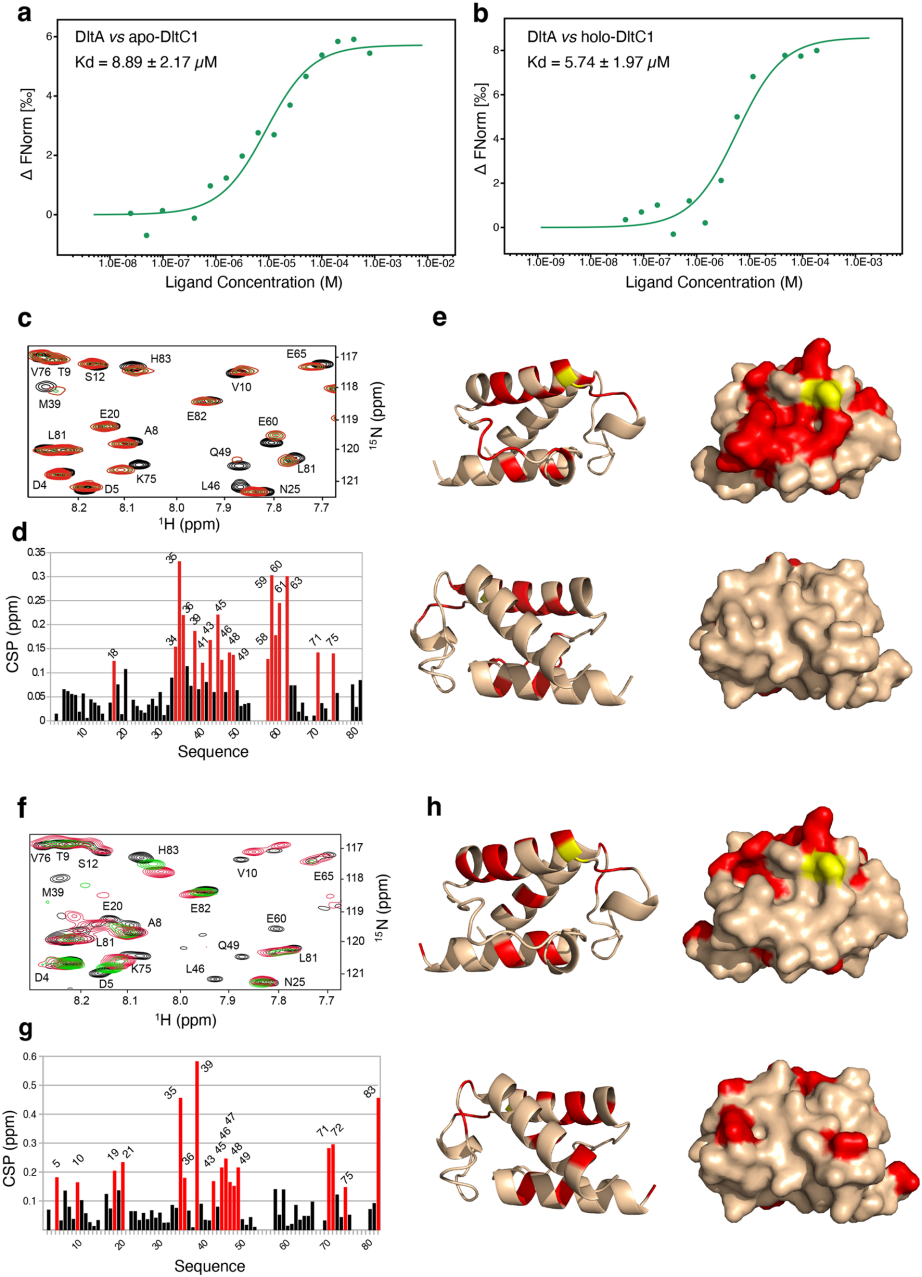
The DltA–DltC1 interaction. (**a** and **b**) MST of the interaction between *L. plantarum* DltA and DltC1. Normalized dose–response curves for the binding interaction between DltA and apo-DltC1 (**a**) and holo-DltC1 (**b**) were obtained by plotting ∆Fnorm against the ligand concentration. The binding curves yield a K_d_ of 8.89 ± 2.17 μM for DltA–apoDltC1 and 5.74 ± 1.97 μM for DltA–holoDltC1. The data are representative of experiments made in triplicate. (**c**–**e**) Comparison of NMR chemical shifts in apo-DltC1 and apo-DltC1:DltA (**c**) Region of the 2D-[^1^H, ^15^N]-BEST-TROSY experiments recorded at 25 °C and pH 6.5 for the ^15^N-labelled apo-DltC1 protein before (black) and after (green and red) addition of unlabelled DltA at a ratio of 1:X and 1:X2, respectively. (**d**) Chemical shift differences calculated from the data displayed in panel (**c**) as the weighted-average distance between the resonance position of the free form of DltA and its equivalent position in the DltC1:DltA complex measured at a ratio of 1:X2 for each residue. Red bars represent the value superior to 2 standard deviations calculated all of the data (CSP > 0.13 ppm). (**e**) CSP superior to 2 standard deviations (> 0.13 ppm as coloured in panel (**d**) are displayed in red on the ribbon and surface representation of DltC1. The bottom panel corresponds to the same orientation as the upper panel but rotate by 180°. (**f**–**h**) Comparison of NMR chemical shifts in holo-DltC1 and holo-DltC1:DltA. (**f**) Region of the 2D-[^1^H, ^15^N]-BEST-TROSY experiments recorded at 25 °C and pH 6.5 for the ^15^N-labelled holo-DltC1 protein before (black) and after (blue, green and red) addition of unlabelled DltA at a ratio of 1:X, 1:X2 and 1:X3, respectively. (**g**) Chemical shift differences calculated from the data displayed in panel (**f**) as the weighted-average distance between the resonance position of the free form of DltA and its equivalent position in the DltC1:DltA complex measured at a ratio of 1:X2 for each residue. Red bars represent the value superior to 2 standard deviations calculated all of the data (CSP > 0.13 ppm). (**h**) CSP superior to 2 standard deviations (> 0.15 ppm as coloured in panel (**g**) are displayed in red on the ribbon and surface representation of Dlt1C. The bottom panel corresponds to the same orientation as bottom panel but rotated by 180°.

The interplay between DltA and DltC allowing the modification of the Ppant-bound DltC with a d-alanyl group brought by DltA remains completely elusive. To further investigate this and test whether DltA can directly interact with DltC1, we performed microscale thermophoresis experiments using purified DltA, apo-DltC1-S38A and holo-DltC1 uniformly modified by Ppant. We observed that apo-DltC1-S38A efficiently interacts with DltA and form a complex with a *K*d of around 10 μM (Fig. 4a). This affinity is not significantly affected, even if slightly higher, when the experiment was performed with holo-DltC1 (Fig. 4b). To confirm these data and to characterize further the DltA–DltC1 interface, we performed NMR titration experiments. For this purpose, we purified both ^15^N-labeled apo-DltC1-S38A and ^15^N-labeled holo-DltC1 and recorded their ^1^H, ^15^N BEST-TROSY spectra alone or in presence of increasing amount of DltA. The chemical shift perturbations (CSP) upon addition of DltA confirmed the DltA/DltC1 interaction (Fig. 4c, d, f and g). In addition, the Ppant group is not essential to the complex formation but seems to rather increase the binding affinity, which is in agreement with the MST results. As the interaction between DltA and DltC from *S. thermophilus* has never been evidenced^23^, our observations therefore provide a first clear characterization of this crucial step of the cytoplasmic pathway of TA d-alanylation.

The CSP were also mapped to the structure of DltC1 to identify the DltA binding sites (Fig. 4e and h). In the case of apo-DltC1, the largest CSP are seen in two main regions ranging from residues 34 to 49 and from 58 to 63. The first one surrounds the catalytic Ser38 and corresponds mainly to the helix α2 (38–52). The second corresponds to the loop connecting α2 and α3 and encompasses the small 3–10 helix observed between residues 58–61. Additional interactions are seen for Thr18 and Lys71 and 75. Likewise, the analysis of the CSP of holo-DltC1 upon DltA addition clearly identified the same region upstream and downstream the catalytic Ser38 (residues 34 to 49), Thr18 and Lys71 and 75. The largest CSP are measured here for Ile35 and Met39 which are also involved in a key hydrophobic interaction with AcpS in the AcpS-DltC1 complex (Fig. 2b). Altogether, these data show that the binding interface of DltC1 to DltA clearly locates on one side of its structure. In addition, they unambiguously reveal that this interface is the same than the one mediating the interaction with AcpS in the AcpS-DltC1 complex described above.

To get insights in the interactions established between DltC1 and DltA, we generated a 3D model structure of the DltA/DltC1 complex (with or without Ppant) with HADDOCK^49^ based on the CSP and the crystal structure of DltC1 and DltA. The best structures with the lowest intermolecular energies showed that DltC1 binds at the interface between the large N-terminal domain and the small C-terminal domain of DltA (Supplementary Fig. 10a and b). In this complex, DltA interacts with DltC1 mainly through H-bonds and ionic interactions involving 3 distinct DltA regions: i) the helix α13 with especially the two Lysine residues 366 and 369 predicted to form a sugar tong locking the beginning of DltC1 α2 helix or the 3–10 helix 58–61, ii) the hinge region between the N-terminal and C-terminal domain (400–405) and iii) the β 23–24 strands of the C-terminal domain (435–440). Importantly, the catalytic Ser38 of DltC1 is surface exposed and covalently bound to the Ppant in front of the substrate binding site of DltA. In addition, the distal end of the Ppant in the extended conformation as described in the AcpS-DltC1 structure (Fig. 2a and Supplementary Fig. 6) would locate in the vicinity of the d-Ala moiety of the adenylate substrate, an ideal position allowing the transfer of d-Ala on the thiol group of the cofactor (Supplementary Fig. 10c and d).

When we superimposed our DltA structure with the generated models, we observed that the P-loop would locate at the level of the Ser38 and the loop before helix α2 (Supplementary Fig. 10e and f). In such a conformation, one can hypothesize that the P-loop could either be involved in DltC1 binding or conflicting with the Ppant position. To validate these structural predictions, we overproduced and purified two DltA mutants: the DltA-**∆**P-loop mutant missing the P-loop region comprised between Thr152 and Lys160 and the DltA-5 M mutant harboring K366A, K369A, D401A, Y435A and K440A substitutions. Interaction assays were performed between DltC1 (apo- or holo-proteins) and DltA-**∆**P-loop or DltA-5 M using MST and NMR titrations experiments (Supplementary Fig. 11). Our results showed that DltA-**∆**Ploop mutant is still able to bind DltC1 with even a lower Kd compared to the wild type protein. This indicates that the P-loop of DltA is not required for the binding to DltC1. Rather, it suggests that the P-loop needs to undergo conformational changes during the catalytic reaction to allow DltC1 binding and Ppant access to the substrate binding site of DltA. Thus, the *Lp*^*NC*8^ strain producing the DltA-**∆**Ploop mutant (Supplementary Fig. 12a) behaves as the ∆*dltA* deletion mutant for both the amount of d-Ala esterified to TAs that is almost undetectable (Supplementary Fig. 12b) and its impact on *Drosophila *growth and development timing that are severely affected (Supplementary Fig. 12c and d) indicating that the P-loop is essential  for the DltA activity.

By contrast, and very importantly, the binding of DltA-5 M to DltC1 was completely abolished. These analyses are in line with our structural predictions (Supplementary Fig. 10) and confirm that the three identified binding regions of DltA are located at the DltA–DltC1 core interface. To validate these observations in vivo, we constructed a *Lp*^*NC*8^ strains producing DltA-5 M (Supplementary Fig. 12a). We measured the levels of d-Ala released from this strain (Supplementary Fig. 12b) and tested its ability to support the growth of *Drosophila* larvae (Supplementary Fig. 12c and d). As expected, mutation of the 5 amino acids of the DltA interaction surface with DltC1 abolished TA d-alanylation as observed with a ∆*dltA* deletion strain and strongly reduced that beneficial impact of *L. plantarum* on the *Drosophila* growth, confirming thus our structural analysis.

## Discussion

In this study we investigated the molecular basis of cytoplasmic components of the d-alanylation pathway of TA, a molecular feature essential for the beneficial action of *L. plantarum* on its animal host growth. We first confirmed that all the proteins involved, the acyl-carrier protein synthase AcpS, the d-alanyl carrier proteins DltC1 and DltC2, the d-alanine ligase DltA and the membrane-bound-O-acetyltransferase DltB are all essential for the d-alanyl esterification of TA and to the beneficial interaction between *L. plantarum* and *Drosophila*. Next, combining different structural and biochemical approaches, we characterized and compared the molecular complexes formed by DltC1 with AcpS, DltA and DltB in order to understand the nature and specificity of these interactions.

**Figure 5:**
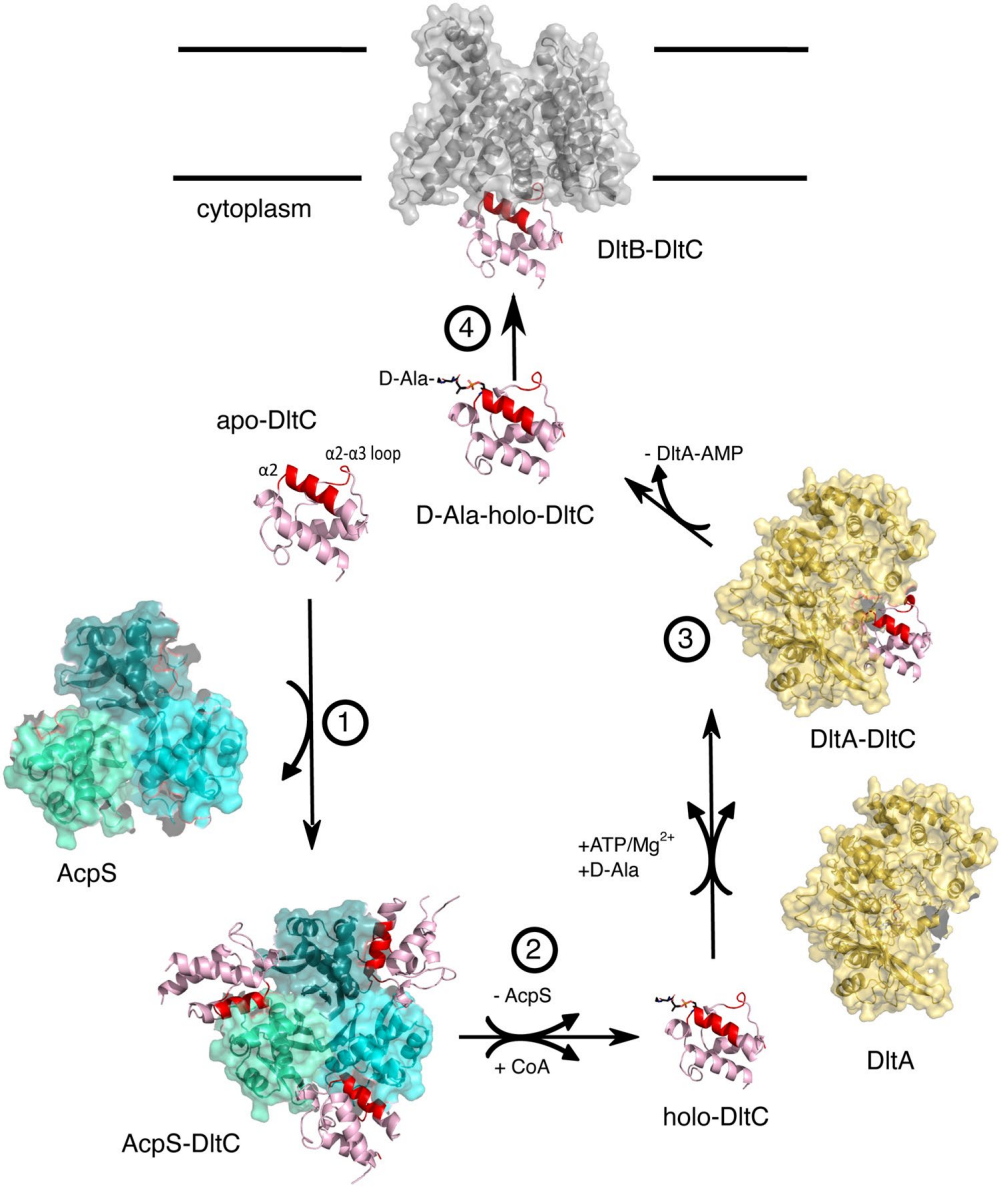
Structural basis of the molecular mechanism of the cytoplasmic steps of the TA d-alanylation pathway. DltC engages its helix α2 and the loop connecting α2 and α3 (binding surface—BS colored in red) to bind the Phosphopantetheinyl transferase AcpS and form a 3:3 stoichiometry AcpS-DltC complex (*step 1*). After the transfer of a CoA molecule by AcpS on the catalytic Ser38 of each DltC molecule, the newly formed holo-DltC detaches from AcpS (*step 2*). Holo-DltC can then interact through the same binding surface (BS colored in red) with DltA in its adenylation state. DltC binds at the interface between the large N-Terminal domain and the small C-terminal domain of DltA and exposes the Ppant moiety covalently bound to Ser38 in front of the binding site of DltA containing a AMP-d-Ala substrate to allow the transfer of the d-alanyl group to the distal end of the Ppant (*step 3*). Finally, DltC harboring the d-Ala-bound Ppant is released from DltA and can bind to DltB with the same binding surface as the one mediating the interaction with DltA and AcpS (*step 4*). The d-Ala-Ppant attached in its extended conformation to DltC will then fit in the DltB funnel to allow the d-Ala transfer in the extracellular space.

We first confirmed the trimeric assembly of the AcpS-DltC1 complex and brought new evidence that two AcpS monomers are required to dock one DltC1 molecule during the catalytic reaction. Our data also newly revealed the extended conformation adopted by the Ppant cofactor after transfer of a CoA molecule by AcpS on the catalytic Serine of DltC1. This conformation differs from the “resting state” observed in the *B. subtilis* AcpS-DltC structure where the Ppant folds back on the DltC protein^24^. Our solution NMR data measured on DltC1 showed as well chemical shifts perturbations of this region in the presence of the Ppant (Supplementary Fig. 14). This demonstrates that the Ppant group can switch between two conformations: the resting state characterized by the interaction of the Ppant at the DltC1 α2-α3 loop surface and an extended conformation. The later conformation provides the molecular insights into the transfer of d-Ala as a thiol ester to the Ppant group by DltA. Importantly, this conformation also confirms the molecular details for the insertion of Ppant into the DltB tunnel^23^. Indeed, as DltB shares 56% sequence identity with DltB from *S. thermophilus*, the structure of the later was used to generate a 3D model of *L. plantarum* DltB and to model the DltB-DltC1 complex (Fig. 5). This model shows that the interaction interface is largely conserved and involves hydrophobic and H-bond interactions formed between the *L. plantarum* DltB residues Met298, Val301, Phe302, Met305 and Arg313 and the DltC1 residues Met39, Val42, Gln 43, Leu46 and Val58. The DltC1 binding interface with DltB appears therefore to be the same as those we characterized with AcpS and with DltA. The residues of the DltC1 interface being highly conserved among homologous DltC sequences (Supplementary Fig. 6), we propose that this surface constitutes a unique interaction surface for all characterized DltC binding partners.

In line with this finding, our data newly showed that DltA and DltC1 form a complex involving the same interaction interface of DltC1 as in the AcpS-DltC1 complex. Although the Ppant cofactor is not required for the interaction between DltC1 and DltA, the binding affinity measured by thermophoresis and NMR titration experiments revealed that it increases the binding affinity. However, this mode of interaction between DltC1 and DltA imposes that DltC1 should dissociate from AcpS first before interacting with DltA. Our finding that the AcpS/DltC1 cannot form a complex when DltC1 is fully Ppanted provides the molecular basis for the dissociation of the AcpS-DltC1 complex allowing subsequent DltC1 binding to DltA. We also propose that the interaction between DltA and DltC1 requires three distinct regions in the N-terminal domain, the hinge region and C-terminal domains of DltA. This mode of interaction allows to dock the DltC1 protein with the catalytic Ser bound to the Ppant cofactor in the vicinity of the substrate binding site of DltA. In this orientation, the thiol extremity of the Ppant is therefore located at the level of the d-Ala consistent with the catalytic reaction. Another important observation concerns the role of the well conserved DltA P-loop. Indeed, beside its key role in the substrate binding, we showed that P-loop undergoes conformational changes that likely occur not only at the early stages of the ATP and d-Ala binding but that could also be required to regulate the interaction with Ppant-DltC1.

Altogether, this study shows that the DltC interaction with AcpS, DltA and finally DltB is sequential and that the formation of the forementioned complexes follows a suite of ordered interactions. The hypothesis raised by Ma et al.^23^ that DltA could load the d-alanyl group on holo-DltC within the DltB-DltC complex is clearly not compatible with our data highlighting the DltC interaction surface common to all binding partners. In addition, our results bring evidence that DltC shows very low plasticity as it does not undergo any significant conformation changes neither upon Ppant attachment and modification nor upon binding to  AcpS, DltA or DltB. DltC would therefore act as an interaction hub for all the successive cytoplasmic steps of the d-Alanylation pathway of the TA.

## Methods

### Drosophila diets, stocks and breeding

*Drosophila* stocks were cultured as described in^30^. Briefly, flies were kept at 25 °C with 12/12 h dark/light cycles on a yeast/cornmeal medium containing 50 g/L of inactivated yeast. The poor yeast diet was obtained by reducing the amount of inactivated yeast to 6 g/L. Germ-free stocks were established as described in^50^. Axenicity was routinely tested by plating serial dilutions of animal lysates on nutrient agar plates. *Drosophila y,*w flies were used as the reference strain in this work.

### Construction of *L. plantarum* strains and growth conditions

Independent markerless *in-frame* deletions on *dltA*, *dltC1*, *dltC2*, and *acpS* genes of *L. plantarum*^*NC8*^ genome were constructed through homology-based recombination with double-crossing over as described by^33^ (Fig. 1a and Supplementary Table 1). Briefly, the 5′- and 3′-terminal regions of each region (in such a way that the two first triplets of the sequence are fused with the two last) were PCR-amplified with Q5 High-Fidelity 2X Master Mix (NEB) from *L. plantarum*^*NC8*^ chromosomal DNA. Primers contained overlapping regions with pG + host9^50^ to allow for Gibson Assembly. PCR amplifications were made using the following primers: OL01/OL02 and OL03/OL04 (*dltA*), OL25/OL26 and OL27/OL28 (*dltB*), OL07/OL08 and OL09/OL10 (*dltC1*), OL13/OL14 and OL15/OL16 (*acpS*), OL19/OL20 and OL21/OL22 (*dltC2*) listed in Supplementary Table 2. The resulting plasmids obtained by Gibson Assembly (NEB) were transformed into *L. plantarum*^*NC8*^ electrocompetent cells and selected at the permissive temperature (28 °C) on MRS plates supplemented with 5 µg/mL of erythromycin. Overnight cultures grown under the same conditions were diluted and shifted to the non-permissive temperature (41 °C) in the presence of 5 µg/mL of erythromycin to select single crossover integrants. Plasmid excision by a second recombination event was promoted by growing integrants at the permissive temperature without erythromycin. Deletions were confirmed by PCR followed by sequencing. The strain deleted for *dltC1* and *dltC2* was obtained by the sequential deletion of *dltC1* followed by *dltC2*.

### Knock-in of dltA modified version in *L. plantarum*^*NC8*^

*L. plantarum*^*NC8*^ strains carrying a modified version of the *dltA* gene was built by knocking-in the modified sequences on *∆dltA* strain constructed in this study. *dltA* modified sequence (*dltA*_∆P-loop_ and *dltA*_*5m*_) were synthetized by Twist Bioscience. In order to perform Gibson Assembly (NEB) the overlapping regions were added by PCR with OL32 and OL33 on the respective plasmid provided by Twist Bioscience. The 5′- and 3′-terminal regions of *dltA* region were PCR-amplified with Q5 High-Fidelity 2X Master Mix (NEB) from *L. plantarum*^*NC8*^ chromosomal DNA using primers OL01/OL31 and OL34/OL4. The 3 fragments were assembled with pG + host9^51^. The resulting plasmids were transformed into *∆dltA* electrocompetent cells and selected at the permissive temperature (28 °C) on MRS plates supplemented with 5 µg/mL of erythromycin. Overnight cultures grown under the same conditions were diluted and shifted to the non-permissive temperature (41 °C) in the presence of 5 µg/mL of erythromycin to select single crossover integrants. Plasmid excision by a second recombination event was promoted by growing integrants at the permissive temperature without erythromycin. *dltA*_*5m*_ and *dltA*_*∆P-loop*_ knock-ins were confirmed by PCR followed by sequencing.

### Quantification of d-alanine by high-performance liquid chromatography (HPLC)

d-alanine esterified to teichoic acids was detected on whole bacterial cells and quantified as described previously by^52^. Briefly, *L. plantarum* wild-type and mutants were grown overnight in 100 ml MRS. Bacteria were harvested, washed twice with 20 mM ammonium acetate, pH 4.7, resuspended in the same buffer, heat-inactivated for 10 min at 100 °C and finally lyophilized. d-alanine was released from whole heat-inactivated bacteria (10 mg) by mild alkaline hydrolysis with 0.1 N NaOH (150 µl) for 1 h at 37 °C. After neutralization with 0.1 N HCl, the extract was incubated with Marfey's reagent (1-fluoro-2,4-dinitrophenyl-5-L-alanine amide; Sigma). This reagent reacts with the optical isomers of amino acids to form diastereomeric *N*-aryl derivatives, which can be separated by HPLC. Separation of the amino acid derivatives was performed on a C_18_ reversed-phase column (Zorbax Eclipse Plus C18 RRHD 2.1 × 50 mm 1.8 µm Agilent) with an Agilent UHPLC 1290 system with a linear elution gradient of acetonitrile in 20 mM sodium acetate buffer (pH 5.0). The eluted compounds were detected by UV absorbance at 340 nm. Quantification was achieved by comparison with d-alanine standards in the range of 50 to 2000 pmol derived with Marfey’s reagent. Mean values were obtained from three independent cultures.

### Larval size measurements

Axenic adults were put overnight in breeding cages to lay eggs on sterile poor yeast diet. Fresh axenic embryos were collected the next morning and seeded by pools of 40 in tubes containing fly food. 1 × 10^8^ CFUs or PBS were then inoculated homogenously on the substrate and the eggs. Petri dishes are incubated at 25 °C until larvae collection. *Drosophila* larvae, 7 days after inoculation, were randomly collected and processed as described by^30^. Individual larval longitudinal length was quantified using ImageJ software^53^.

### Developmental timing determination

Axenic adults were placed in breeding cages overnight to lay eggs on sterile poor-yeast diet. Fresh axenic embryos were collected the next morning and seeded by pools of 40 in tubes containing fly food. A total of 1 × 10^8^ CFUs of each strain or PBS was then inoculated homogeneously on the substrate and the eggs and incubated at 25 °C. The emergence of pupae was scored every day until all pupae had emerged. D50 (day when 50% of the pupae emerged) was determined using D50App^33^.

### *Escherichia coli* plasmid construction

DNA fragments were amplified by polymerase chain reaction using *L.plantarum* cDNA as a template and oligonucleotides listed in Supplementary Table 3. The DNA encoding the full-length wild-type *L. plantarum* DltC1, and the DltC1-S38A mutant were cloned into the NcoI and XhoI sites of the pET-28a(+) vector that expresses proteins fused to a C-terminal hexahistidine (His)6 tag (Supplementary Table 4). To construct plasmids producing the full-length wild-type *L. plantarum* AcpS, the full-length wild-type *L. plantarum* DltA and the DltA-∆P-loop mutant (missing the loop region comprised between T152 and K160), the corresponding DNA fragments were cloned into the NdeI and PstI sites of the pT7-7 vector that expresses proteins fused to a TEV (tobacco etch virus) cleavage site and a C-terminal Hexahistidine (His)_6_ tag. An untagged *Lp*AcpS^WT^ construct was also prepared in the pT7-7 vector by introducing a stop codon before the TEV cleavage site and the C-terminal hexahistidine tag (Supplementary Table 4).

The gene of the DltA-5 M harboring the K366A, K369A, D401A, Y435A and K440A mutations was synthesized by Twist Bioscience in a pET-28a(+) vector that contains a a TEV (tobacco etch virus) cleavage site followed by a C-terminal Hexahistidine (His)_6_ tag (Supplementary Table 4). The nucleotide sequences encoding the wild type DltC1 fused to a C-terminal His6 tag and the wild type AcpS were also inserted in pRSF-Duet1 vector for co-expression (Supplementary Table 4). The final constructs were verified by DNA sequencing.

### Protein production and purification

DltA, DltA-∆P-loop, DltA-5 M, untagged and C-terminal 6His tagged AcpS, and the DltC1-6His-AcpS construct cloned in pRSF-Duet1were expressed in *E. coli* BL21 (DE3) cells. DltC1 and DltC1-S38A mutant were expressed in *E. coli* BL21 (DE3)-RIPL cells. Cells were grown in LB media at 37 °C and induced with 0.5 mM isopropyl b-d-1-thiogalactopyranoside (IPTG) overnight at 18 °C. The cells were then harvested by centrifugation and resuspended in lysis buffer (50 mM Tris, pH 7.5, 500 mM NaCl, 10% Glycerol, 1 mM Dithiothreitol (DTT) 0.01 mg/ml Lysozyme, 0.006 mg/ml Dnase/RNase, 1 × antiprotease CLAPA). The resuspended cells were disrupted by sonication and centrifuged at 14,000 g for 45 min. Proteins were all purified by a first step of Ni–NTA affinity chromatography with the elution buffer (50 mM Tris, pH 7.5; 300 mM NaCl; 1 mM Dithiothreitol (DTT); 250 mM Imidazole). The eluted fractions were then concentrated and buffer-exchange was performed in a centrifugal filter unit with gel filtration buffer (50 mM Tris, pH7.5; 100 mM NaCl and 1 mM Dithiothreitol (DTT)). The proteins were finally applied to a Superdex 200 10/300 GL size exclusion column (GE Healthcare) and eluted with gel filtration buffer.

In order to obtain the AcpS-DltC1 complex, the DltC1 containing fractions eluted from the first Ni–NTA chromatography were mixed with lysate from cells overexpressing untagged AcpS. The mix was concentrated and buffer-exchange was performed in a centrifugal filter unit with lysis buffer. The protein mix was loaded onto a nickel-NTA (Qiagen) column and recovered with elution buffer. The eluted fractions (containing DltC1 and AcpS-DltC1 complex) were then concentrated and buffer-exchange was performed in a centrifugal filter unit with gel filtration buffer. The sample was applied to a Superdex 200 10/300 GL size exclusion column and eluted with gel filtration buffer. Isotopically labelled proteins for NMR spectroscopy were prepared by growing cells in M9 minimal media containing ^15^NH_4_Cl and/or [^13^C]-glucose. Purifications were performed as described above except that the gel filtration buffer was replaced by an NMR buffer composed of 20 mM MES, pH6.5; 150 mM NaCl.

### Crystallization, data collection and structure determination

Crystallization screenings were performed by the sitting-drop vapor-diffusion method at 293 K using crystallization kits Crystal Screen 1 and 2, PEG/Ion PEG/Ion 2 (Hampton Research). The crystallization drops (0.2µL protein solution and 0.2µL reservoir solution) were set up using a Mosquito crystallization robot. The DltA wild type protein was concentrated to 24 mg/ml and mixed with 1 mM ATP and 1 mM d-Ala prior to crystallization. Diffraction quality crystals grew after around 3 months from a solution of the PEG Ion crystallization kit containing 20% PEG3350 and 0.2 M KCl. The AcpS/DltC1 complex was concentrated to 4.8 mg/ml and crystallized in 3 days in one of the PEG Ion crystallization kit conditions containing 20% PEG3350 and 0.2 M Sodium Malonate. After rapid soaking in mother liquor supplemented with 20% (v/v) glycerol, crystals were flash-cooled in liquid nitrogen prior to diffraction experiments. Diffraction data were collected at cryogenic temperature (100 K) on beamline ID30A-3 at the European Synchrotron Radiation Facility (ESRF, Grenoble, France) from the AcpS/DltC1 complex crystals and on beamline PROXIMA-1 at SOLEIL synchrotron (Gif sur Yvette, France) for the DltA crystals.

Data were processed using the XDS package^54^. The structures were solved by molecular replacement using Phaser implemented in PHENIX^55^. The PDB entries 3DHV and 1F80 were used as starting models respectively to solve DltA and the AcpS/DltC1 complex. Both structures were refined using iterative rounds of COOT^56^ and PHENIX^55^. The quality of the final structure was assessed with MOLPROBITY before deposition at the PDB under the code 7R27 (DltA) and 7R49 (AcpS-DltC1). Sequence alignments and structure images were generated with PyMOL (Schrödinger, LLC), ESPript and ENDscript^57^.

### NMR resonance assignments

The 2D- and 3D-NMR experiments were collected on a 3 mm NMR tube filled with 150 µL of a 268 μM ^13^C, ^15^N-labeled apo-DltC1 (S38A) sample prepared in 20 mM HEPES, 150 mM NaCl buffer at pH 6.5 and containing 7%D_2_O. Backbone resonance assignments were carried out using a combination of 2D ^1^H-^15^N-BEST-TROSY and 3D BEST-HN(CO)CACB, BEST-HNCACB, BEST-HNCO, BEST-HN(CA)CO, BEST-H(NCACO)NH^58,59^. A 2D ^1^H-^15^N-BEST-TROSY were acquired on a 150 μM ^15^N-labeled holo-DltC1 prepared in the same buffer and was assigned by comparison with the data acquired on ^13^C, ^15^N-labeled apo-DltC1 sample. All the spectra were acquired at 25 °C on Bruker Avance III 700 MHz spectrometer equipped with a xyz-gradient a TCI cryoprobe. The NMR spectra were processed using the TopSpin™ software by Bruker in its 4.1 version and were analyzed using the CcpNmr Analysis software in its version 3.0^60^. The ^1^H chemical shifts were referenced to the internal standard 4,4-dimethyl-4-silapentane-1- sulfonic acid (DSS) methyl resonance. ^13^C and ^15^N chemical shifts were referenced indirectly using the IUPAC-IUB protocol^61^.

### NMR titration experiments

Interaction studies of apo-DltC1 and holo-DltC1 with DltA, DltA-5M and DltA-**∆**P-loop were performed using 3 mm NMR tubes with ^15^N-labeled apo-DltC1 and holo-DltC1 at concentration varying from 150 to 300 μM and prepared in a buffer containing 20 mM HEPES buffer, pH 6.5, 150 mM NaCl and 7% (vol/vol) D_2_O. Unlabelled DltA samples were prepared in the same buffer at a concentration of 342 μM for titration with apo-DltC1 and 430 μM for titration with holo-DltC1 titration. DltA-5M and DltA-**∆**P-loop mutants were also prepared in the same buffer at a concentration of 781 μM and 139 μM, respectively. Unlabeled DltA proteins were successively added to the ^15^N-labelled DltC1 proteins to reach a protein-to-protein ratio of 0, 0.5, 1 and 1.3 for DltA/apo-DltC1, a ratio of 0, 0.2, 0.4, 0.6, 0.8, 1.0 and 1.2 for DltA/holo-DltC1, a ratio of 0, 0.2, 0.4, 0.6, 0.8, 1.0 and 1.8 for DltC1/DltA-5 M and holo-DltC1/DltA-5M and a ratio of 0, 0.1, 0.2, 0.5, 1.0 and 1.8 for holo-DltC1/DltA-**∆**P-loop. [^1^H, ^15^N]-BEST-TROSY spectra were collected at 25 °C for each protein ratio using Bruker AVANCE spectrometers equipped with a TCI cryoprobe and operating at 700 MHz proton frequency. Analysis software CcpNmr 3.0 was used to monitor protein chemical shift perturbations for every assigned amide resonance by superimposition of the ^15^N-BEST-TROSY spectra and automatic peak picking. Chemical shift perturbations (Δδ) were calculated on a per-residue basis for the highest substrate-to-protein ratio as described previously^62^.

### Docking

Models of holo-DltC1 in complex with DltA were built with the version HADDOCK2.2 of “The HADDOCK web server for data-driven biomolecular docking”^49^. As starting structures, we used the X-ray crystallography structure of *L. plantarum* holo-DltC1 (this study, PDB ID 7R49), a model of full length DltA obtained with Phyre^63^ and the substrate position from the DltA N-terminal domain structure in complex with d-Ala-AMP (this study, PDB ID 7R27). Residues of DltC1 that showed chemical shift perturbations above the threshold in Fig. 4g were considered as active Ambiguous Restraints (AIR). Calculations were performed with 2000 structures during the HADDOCK rigid body energy minimization, 200 structures during the refinement, and 200 structures during the refinement in explicit water. The output model structures were sorted with the HADDOCK built-in clustering tool using the Fraction of Common Contacts (FCC) method^64^ with a 0.60-Å cutoff and a minimum of 4 structures per cluster. To improve the convergence during the HADDOCK run, a 2.5-Å unambiguous restraint was introduced between the sulfur atom of the Ppant and the phosphorus of the AMP molecule.

### Microscale thermophoresis assays

Protein–protein interactions were analyzed by microscale thermophoresis (MST)^65^. Buffer of purified and concentrated protein samples was exchanged on a desalting PD-10 column to labeling buffer containing Hepes 25 mM pH7.5, NaCl 300 mM, Tween20 0.05% (w/v). Proteins were then labeled with NHS red fluorescent dye according to the instructions of the RED-NHS Monolith NT Protein Labeling kit (NanoTemper Technologies GmbH, Munchen, Germany). After a short incubation of target-partner complex, the samples were loaded into MST standard-treated glass capillaries and measurements were performed at 22 °C. The assays were repeated three times for each affinity measurement. Data analyses were performed using NanoTemper Analysis software provided by the manufacturer.

### Statistics and reproducibility

Data representation and analysis of d-alanine quantification and *Drosophila* larval size measurements and developmental timing determination were performed using Graphpad PRISM 6 software (www.graphpad.com). A total of 3 to 5 replicates were used for all experiments performed in this study in order to ensure representativity and statistical significance. All samples were included in the analysis. Experiments were done without blinding. Two-sided Mann Whitney’s test was applied to perform pairwise statistical analyses between conditions for *Drosophila* larval size measurements and developmental timing determination experiments.

All MST assays were performed in triplicate and K_d_ represent the mean values with corresponding standard deviations.

## Supplementary Information

Supplementary Information.

## Data Availability

The datasets generated and/or analyzed during the current study are available in the Worldwide Protein Data Bank (www.pdb.org). Coordinates and structure factors have been deposited at wwPDB under the accession code 7R27 (DltA) and 7R49 (AcpS-DltC1). Other data supporting the findings of this study are available from the corresponding authors upon reasonable request.
