# Supplementary material for: DltC acts as an interaction hub for AcpS, DltA and DltB in the teichoic acid d-alanylation pathway of Lactiplantibacillus plantarum
Source: Sci Rep. 2022 Jul 30;12:13133. doi: 10.1038/s41598-022-17434-2 (PMC9338922; doi:10.1038/s41598-022-17434-2)
Supplement: Supplementary file 1 — Supplementary Information. [file 41598_2022_17434_MOESM1_ESM.pdf]

Supplementary information

DltC acts as an interaction hub for AcpS, DltA and DltB in the teichoic acid D-alanylation pathway of *Lactiplantibacillus plantarum*

Nikos Nikolopoulos[1], Renata C. Matos[2], Pascal Courtin[3], Isabel Ayala[4], Houssam Akherraz[2], Jean-Pierre Simorre[4], Marie-Pierre Chapot-Chartier[3], François Leulier[2], Stéphanie Ravaud[1,*] and Christophe Grangeasse[1,*]

[1] Molecular Microbiology and Structural Biochemistry, CNRS UMR 5086, Université Claude Bernard Lyon 1, Lyon, France.

[2] Institut de Génomique Fonctionnelle de Lyon, École Normale Supérieure de Lyon, CNRS UMR 5242, Université Claude Bernard Lyon 1, Lyon, France.

[3] Université Paris-Saclay, INRAE, AgroParisTech, Micalis Institute, 78350, Jouy-en-Josas, France

[4] Institut de Biologie Structurale, Université Grenoble Alpes, CEA, CNRS UMR 5075, 3800, Grenoble, France

* Correspondence: Stéphanie Ravaud (stephanie.ravaud@ibcp.fr) and Christophe Grangeasse (christophe.grangeasse@ibcp.fr)

Supplementary Table 1. Bacterial strains used in this study.

Strain	Relevant characteristics	Reference or source
E. coli		
DH5a	F^- *endA1 glnV44 thi-1 recA1 relA1 gyrA96 deoR nupG purB20* φ80d*lacZ*ΔM15 Δ(*lacZYA-argF*)U169, hsdR17($r_K^- m_K^+$), λ^-	Laboratory collection
BL21(DE3)	F^- *ompT gal dcm lon* $hsdS_B(r_B^- m_B^-)$λ(DE3 [*lacI lacUV5-T7p07 ind1 sam7 nin5*]) [$malB^+$]$_{K\text{-}12}$(λ^S)	Novagen
BL21-CodonPlus (DE3)-RIPL	F–ompT hsdS(rB– mB–) dcm+ Tetr galλ(DE3) endA Hte [argU proL Camr] [argU ileY leuW Strep/Specr]	Novagen
TG1	*supE hsd5h thi (Δlac-proAB) F' (traD36 proAB-lacZΔM15)*	[66]
GM1674	$dam^- dcm^-$ $repA^+$	[67]
L. plantarum		
NC8	Isolated from grass silage, plasmid free	[68]
Δ*dltA*	NC8 strain deleted for *nc8_1736*	This study
Δ*dltB*	NC8 strain deleted for *nc8_1735*	This study
Δ*dltC1*	NC8 strain deleted for *nc8_1734*	This study
Δ*dltC2*	NC8 strain deleted for *nc8_1214*	This study
Δ*dltC1C2*	NC8 strain deleted for *nc8_1734* and *nc8_1214*	This study
Δ*acpS*	NC8 strain deleted for *nc8_0463*	This study
DltA5m	Knock-in of a modified version of *dltA* gene harboring the pentamutation in Δ*dltA* strain	This study
$DltA_{\Delta P\text{-loop}}$	Knock-in of a modified version of *dltA* gene lacking the P-loop region in Δ*dltA* strain	This study

Supplementary Table 2. Primers used for the construction of *L. plantarum* strains.

Name	Sequence (5'→3')*	Reference
OL01	AGTGGATCCCCCGGGCTGCAGGAATTTATAATTATTCATCCGT	This study
OL02	AAAGAAATCATTCAATCATAATTAAAACTCCCCCCA	This study
OL03	TTTAATTATGATTGAATGATTTCTTTTGACCCATATGGT	This study
OL04	CTTGATATCGAATTCCTGCATCGGAGTTTCAACACCCATCA	This study
OL05	AACGCTTCGCCAAATGTTAG	This study
OL06	TGATCCGGCTCTTAAAGACC	This study
OL07	TGGATCCCCCGGGCTGCAAAGGATTACGATAGTGTCCCGAC	This study
OL08	CGTTCATTGTGTCATTACGCTCCCTTATTGT	This study
OL09	GTAATGACACAATGAACGTTGCCAAACGC	This study
OL10	TGATATCGAATTCCTGCAACTGTTCGAGTCCGTTTGCG	This study
OL11	AGAATTCCATCCGGTACTG	This study
OL12	CTGGTGACTTCGTGTAATTC	This study
OL13	TGGATCCCCCGGGCTGCACGCGTCATCTCAAATACGAGT	This study
OL14	CCATCACAAAATCACGCTGTGACCCCCTC	This study
OL15	AGCGTGATTTTGTGATGGTTGTAATTGGGGAG	This study
OL16	TGATATCGAATTCCTGCACGTCAAGCGTGCCGTTAG	This study
OL17	AGTCGGCAGATGTTCGTATG	This study
OL18	TTGTAATCGGCGTTCATAGC	This study
OL19	TGGATCCCCCGGGCTGCAGTCATGAGTGCCGAGCGAC	This study
OL20	TTTTATTTATTGGATCAAAATACGCCCCTTATCA	This study
OL21	CGTATTTTGATCCAATAAATAAAAATTCAAATCGCCGT	This study
OL22	TGATATCGAATTCCTGCAGGACTGAGTGAAATAGGTAAATCCC	This study
OL23	CCATTCAGGGACACCATGCC	This study
OL24	ATACAACCATGTGAATTGGC	This study
OL25	TGGATCCCCCGGGCTGCATTACCAATCAACGTTTGGGTCTC	This study
OL26	TTATATTATGGAATCATTCTGGGTTAACCTCT	This study
OL27	CCAGAATGATTCCATAATATAATTACAATAAGGGAGCGT	This study

OL28	TGATATCGAATTCCTGCAAGCTGCCACACGTGCC	This study
OL29	ATTTGACCTATCCGTGATGG	This study
OL30	CTGGTAACTTGTTAGCTTGC	This study
OL30	CTGGTAACTTGTTAGCTTGC	This study
OL31	GTTCTTAATCATAATTAAAACTCCCCCCAAACA	This study
OL32	GGAGTTTTAATTATGATTAAGAACATCATTACAACGA	This study
OL33	TCAAAAGAAATTATTCTGGGTTAACCTCTTT	This study
OL34	CCCAGAATAATTTCTTTTGACCCATATGGTAACCC	This study

*Overlapping sequences for Gibson assembly are underlined.

Supplementary Table 3. Primers used for *E. coli* plasmid constructions.

Number	Name	Sequence (5' to 3')	Reference
1	5-pDltA	CATGCATATGATTAAGAACATCATTACAACG	This study
2	3-pDltA	CGCTGCAGTTCTGGGTTAACCTCTTTAATGATCG	This study
3	5-pDltC	AGATATACCATGGCTACAATGGATGATACAAAAGCAACGG	This study
4	3-pDltC	TCGACTCGAGTTGCAAGTTCTCAACCTTTGCAAC	This study
5	5- p-apo-DltC1	GAATCTTAGATGCAATGGGTTCC	This study
6	3- p-apo-DltC1	GGAACCCATTGCATCTAAGATTC	This study
7	5-pAcpS	GGAGATATACATATGGTGATTTATGGTACC	This study
8	3- pAcpS	ATCGATAAGCTTTTACAAATTGCCTCTTTC	This study
9	5-pAcpS_6His	CACCACCACCACCACCACTAAAAGCTTATCGATGATAAGCTGTCAA	This study
10	3-pAcpS_6His	GTGGTGGTGGTGGTGGTGCAAATTGCCTCTTTCCAATATAACTTGT	This study
11	3-pDltC/AcpS	CACCACCACTAATGCTTAAGTCGAAC	This study
12	5-pRSFDuet	TAATGCTTAAG	This study
13	3-pRSFDuet_6His	CTTAAGCATTAGTGGTGGTGGTGGTGGTGC	This study
14	5-pRSFDuet_NdeI	CATATGTATATCTCC	This study
15	3-pRSFDuet_T7.7	TAAAAGCTTATCGATTTAACCTAGGCTGCTG	This study
16	5- pDltA-ΔP-loop	TTATCTTTGGGGTCCAAATTAGTCATGATAACTTGG	This study
17	3- pDltA-5M	TTTGGACCCCAAAGATAATATAGTAATTATCATCGC	This study

Supplementary Table 4. Plasmids used in this study

Plasmid	Description and main characteristics	Source	Primers
pG+host9	Erm^r, repATs	[52]	-
pET-28a(+)	T7 promoter, C-terminal 6×His, Kan^R	Novagen	-
pT7-7	T7 promoter, C-terminal 6×His, Amp^R	Novagen	-
pRSF-Duet1	Two MCS, Two T7 promoters, Kan^R	Novagen	-
pDltA	pT7-7 derivative encoding *L. plantarum* full-length wild-type DltA fused to a tobacco etch virus (TEV) cleavage site and a C-terminal $(His)_6$ tag	This study	1,2
pDltC1	pET-28a(+) derivative encoding *L. plantarum* full-length wild-type DltC1 fused to a C-terminal $(His)_6$ tag	This study	3,4
p-apo-DltC1	pET-28a(+) derivative encoding *L. plantarum* DltC1 S38A mutant fused to C-terminal $(His)_6$ tag	This study	5,6
pAcpS	pT7-7 derivative encoding *L. plantarum* full-length wild-type AcpS	This study	7,8
pAcpS_6His	pT7-7 derivative encoding *L. plantarum* full-length wild-type AcpS fused to C-terminal $(His)_6$ tag	This study	9,10
pDltC1/AcpS	pRSF-Duet1 derivative encoding *L. plantarum* full-length wild-type DltC1 fused to a C-terminal $(His)_6$ and *L. plantarum* full-length wild-type AcpS	This study	3,11,12,13,5,14,15
pDltA-ΔP-loop	pT7-7 derivative encoding *L. plantarum* DltAΔP-loop mutant fused to a TEV cleavage site and a C-$(His)_6$ tag	This study	16,17
pDltA-5M	pET-28a(+) derivative encoding *L. plantarum* DltA-5M mutant fused to a C-terminal $(His)_6$ tag	This study	-

Supplementary References

66. Baer, R. *et al.* DNA sequence and expression of the B95-8 Epstein—Barr virus genome. *Nature* **310**, 207–211 (1984).

67. Palmer, B. R. & Marinus, M. G. The dam and dcm strains of Escherichia coli — a review. *Gene* **143**, 1–12 (1994).

68. Axelsson, L. *et al.* Genome sequence of the naturally plasmid-free Lactobacillus plantarum strain NC8 (CCUG 61730). *J Bacteriol* **194**, 2391–2392 (2012).

Supplementary Figure 1

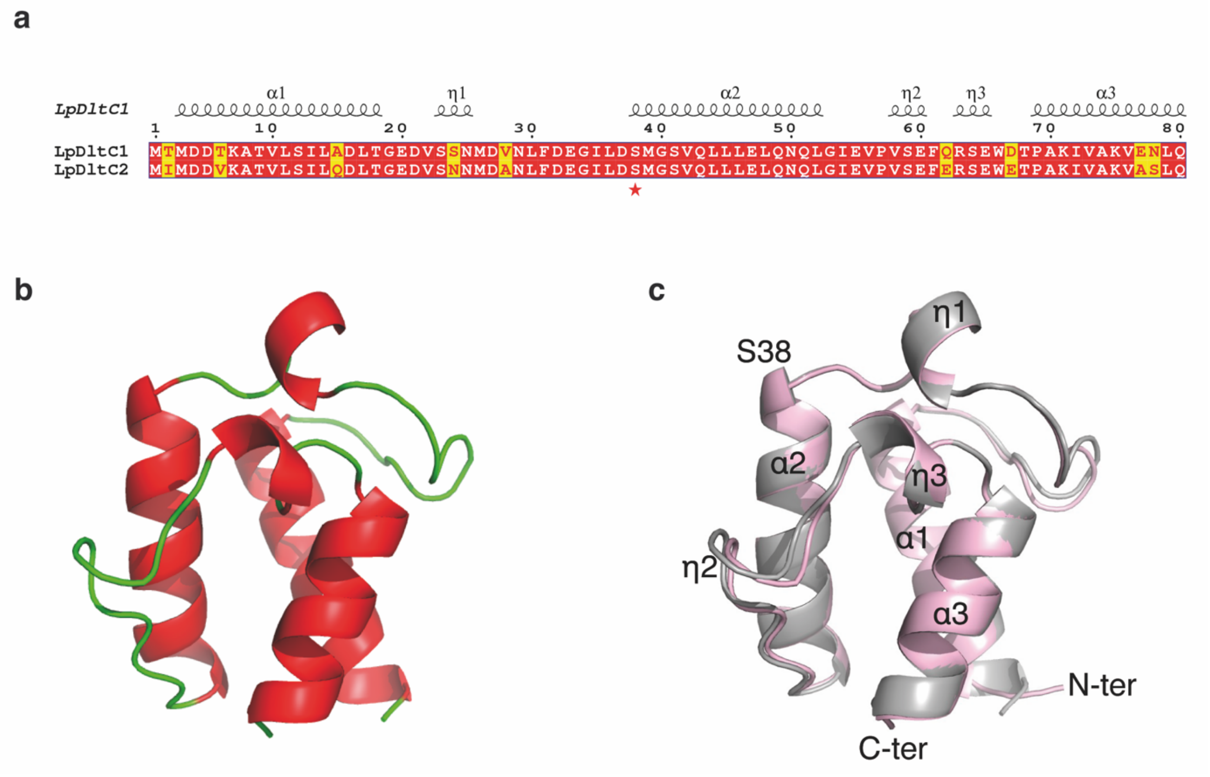

Supplementary Figure 1. The *L. plantarum* DltC1 and DltC2 proteins. (**a**) Sequence alignment between DltC1 and DltC2 of *L. plantarum*. The secondary structure of DltC1 extracted from the X-ray structure of the AcpS-DltC1 complex is depicted above (this study, PDB entry 7R49). The position of the catalytic Ser38 is highlighted by a red star. The figure was generated by ESPript (https://espript.ibcp.fr/). (**b**) Cartoon representation of DltC2 structure. The 3D structure was constructed by homology modeling using the X-ray structure of DltC1 as a template with the help of Swiss Model (https://swissmodel.expasy.org). (**c**) Superimposition of the 3D structures of DltC1 in pink and DltC2 in grey

Supplementary Figure 2

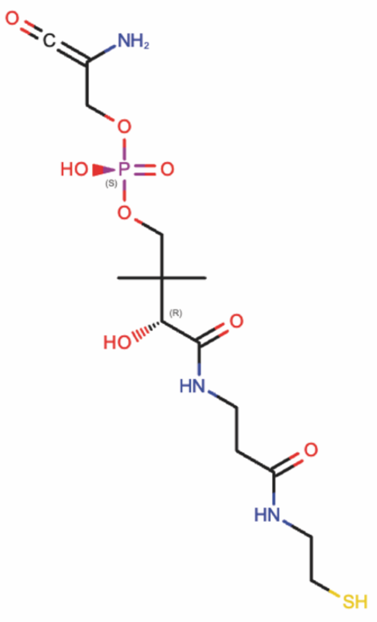

Supplementary Figure 2. The Ppant cofactor. Structure of the 4'-(3-amnopropionic) Phosphopantetheine, Ppant (PDB chemical ID PN2)

Supplementary Figure 3

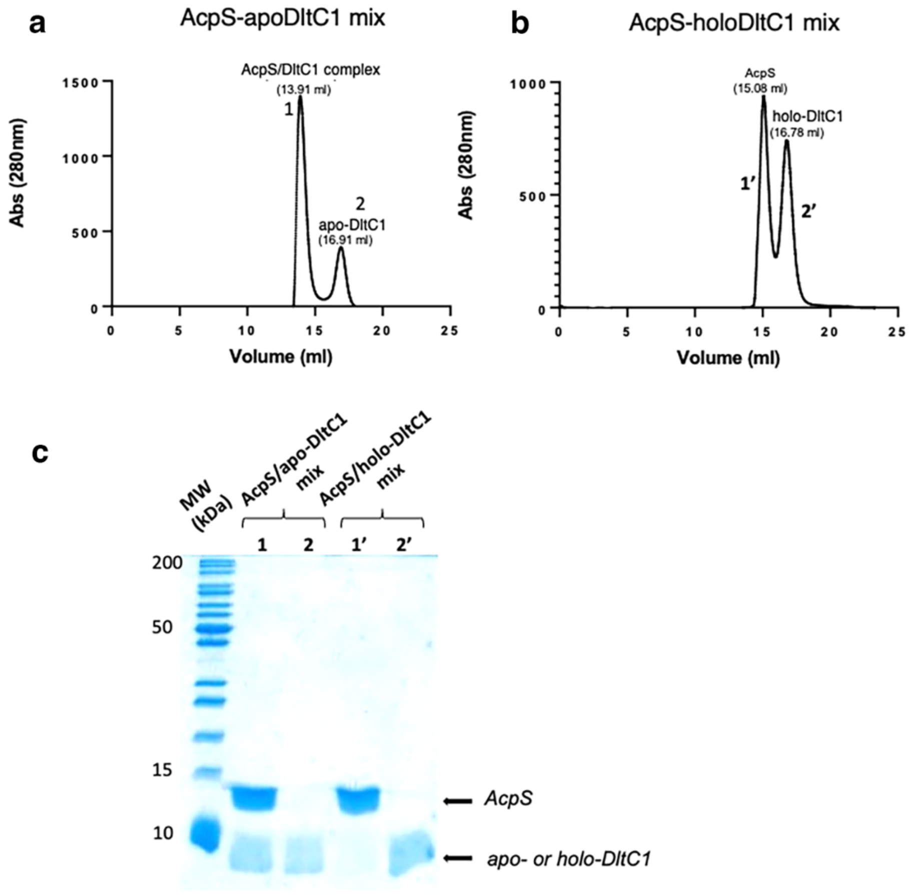

Supplementary Figure 3. Size exclusion chromatography (SEC) and SDS PAGE analysis of the AcpS-DltC complex formation. (**a**) Elution profile after injection of the mixture of Ni-affinity purified AcpS and apo-DltC1 (S38A) (molar ration 1:1.11) on a Superdex 200 10/300 GL. As confirmed by the SDS PAGE shown on (c), the first peak contains the AcpS-DltC1 complex while the second peak corresponds to the excess of free DltC1. (**b**) Elution profile after injection of the mixture of Ni-affinity purified AcpS and holo-DltC1 (molar ration 1:1.11) on a Superdex 200 10/300 GL. As confirmed by the SDS PAGE shown on (c), the first peak contains the free AcpS while the second peak corresponds to holo-DltC1. (**c**) SDS PAGE of the eluted fractions from SEC-a and SEC-b. The bands corresponding to AcpS (14.1 kDa) and DltC1 (9.9 kDa) are shown.

Supplementary Figure 4

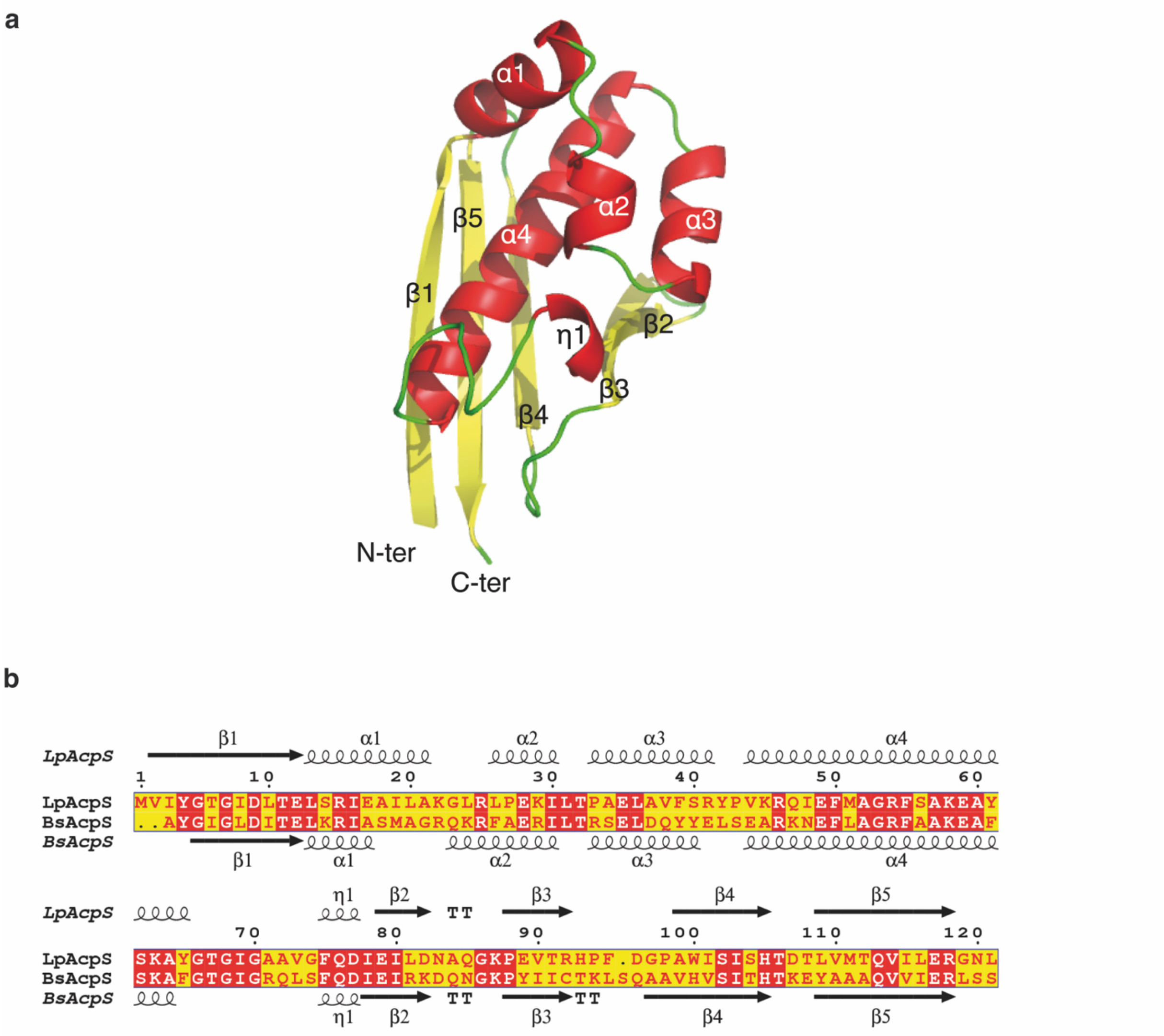

Supplementary Figure 4. 3D structure of AcpS from *L. plantarum*. (**a**) Cartoon representation of the LpAcpS structure from the AcpS-DltC1 complex (this study, PDB entry 7R49). (**b**) Sequence alignment between *L. plantarum* AcpS *and B. subtilis* AcpS. The respective secondary structures extracted from the X-ray structures of the AcpS-DltC complexes are depicted above and below (This study and PDB entry 1F80). The figure was generated by ESPript (https://espript.ibcp.fr/).

Supplementary Figure 5

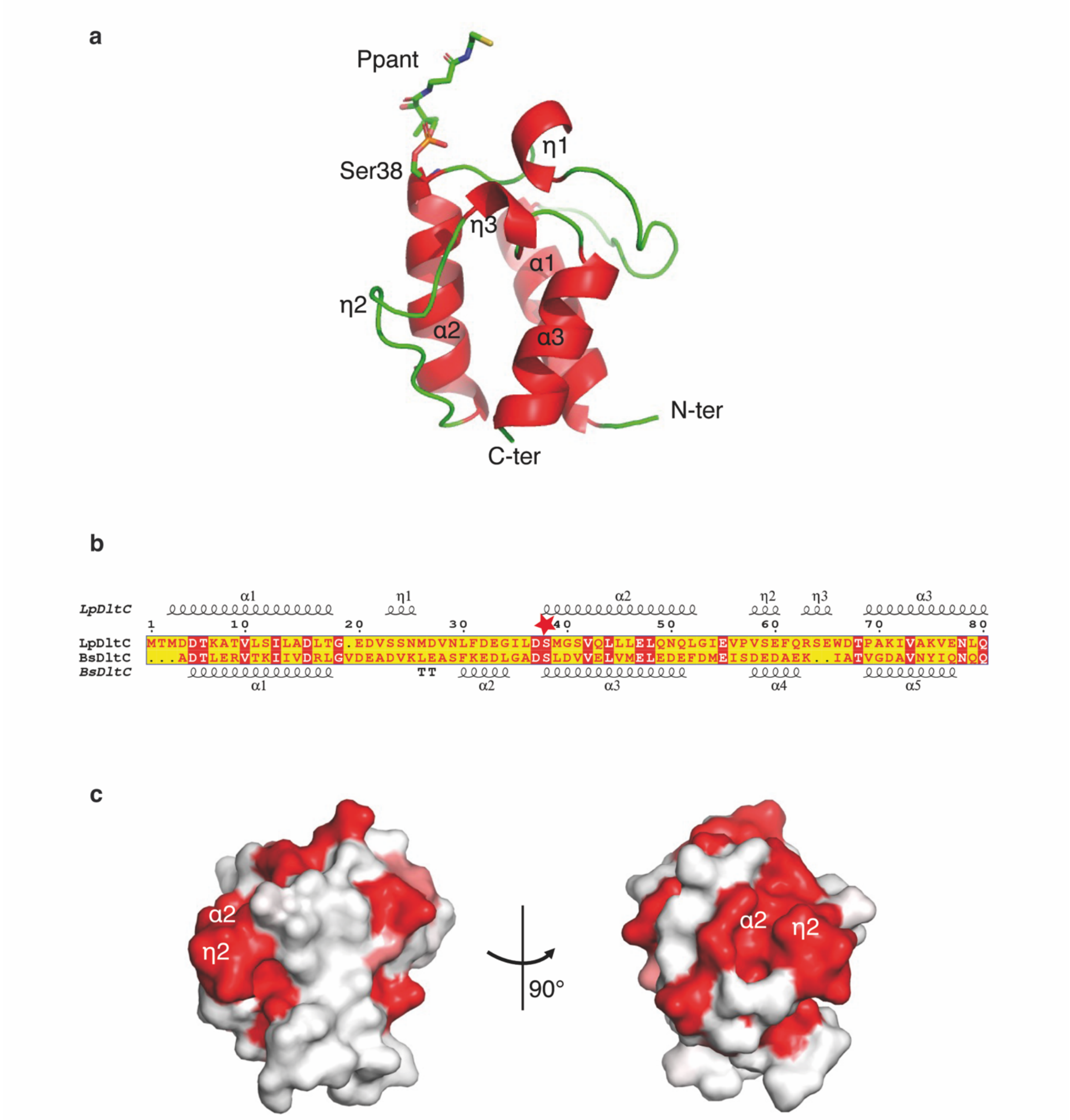

Supplementary Figure 5. 3D structure of DltC1 from *L. plantarum.* (**a**) Cartoon representation of the DltC1 structure from the AcpS-DltC1 complex (this study, PDB entry 7R49). (**b**) Sequence alignment between *L. plantarum* DltC1 and *B. subtilis* DltC (ACP). The respective secondary structures extracted from the X-ray structures of the AcpS-DltC complexes are depicted above and below (This study and PDB entry 1F80). The figure was generated by ESPript (https://espript.ibcp.fr/). (**c**) Surface representation of DltC1 according to sequence conservation. The color ramping from white (low score) to red (identity) locates areas of weak and strong sequence conservation. The figure on the right is in the same orientation as in a, the figure on the left was rotated by 90°. The DltC binding interface (α2-η2) is indicated. The figure was generated by ENDScript (https://endscript.ibcp.fr/).

Supplementary Figure 6

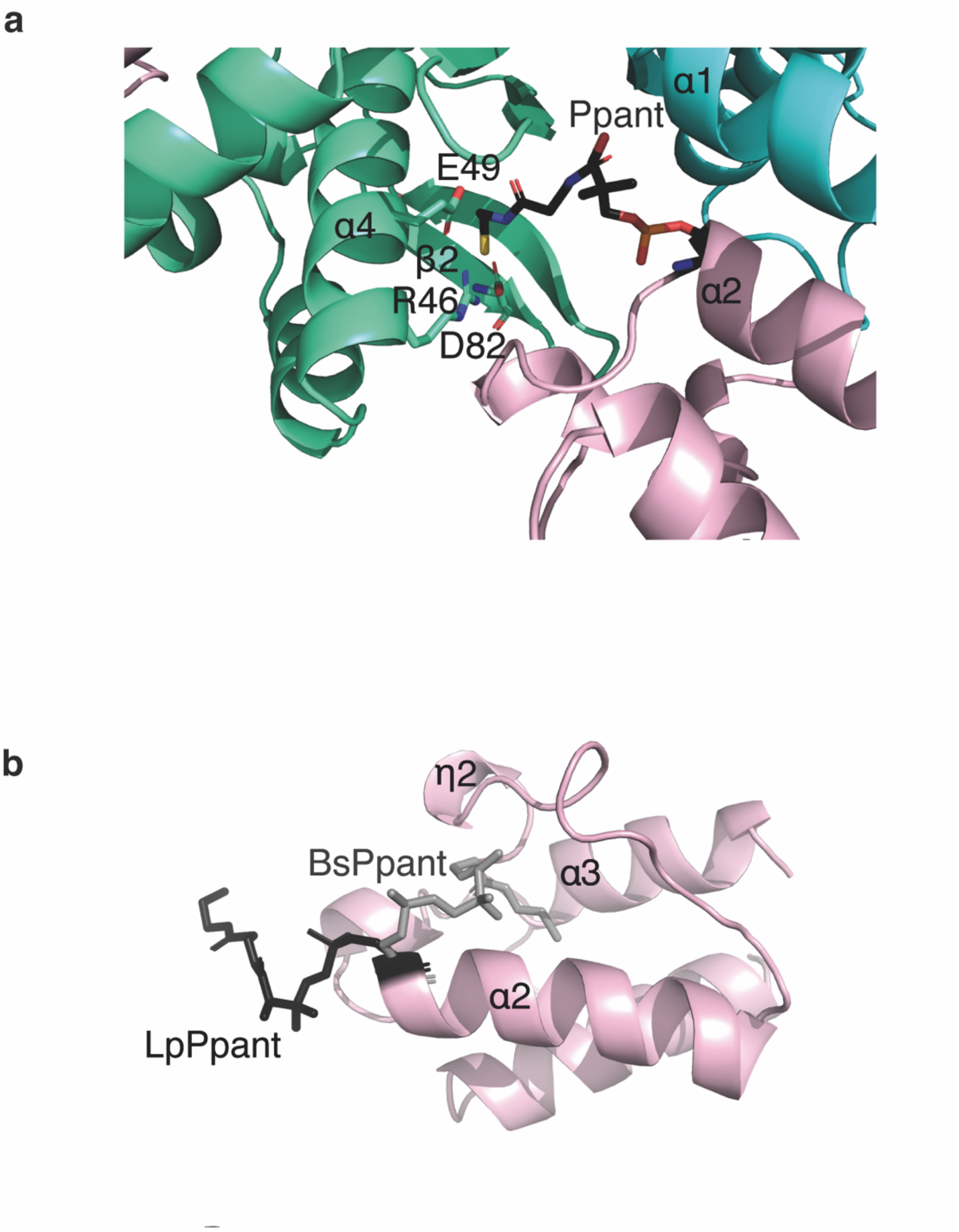

Supplementary Figure 6. The Ppant conformation. (**a**) The Ppant (in black) bound to one DltC1 monomer (in pink) is observed in an extended conformation in the AcpS-DltC1 structure from *L. plantarum* at the interface between two AcpS monomers shown in cyan and green (this study, PDB entry 7R49). The AcpS residues interacting with the thiol end of the Ppant are shown in stick. (**b**) Comparison of the Ppant conformations, extended in the AcpS-DltC1 structure from *L. plantarum* (in black) and resting in the AcpS –DltC structure from *B. subtilis* (in grey – PDB entry 1F80).

Supplementary Figure 7

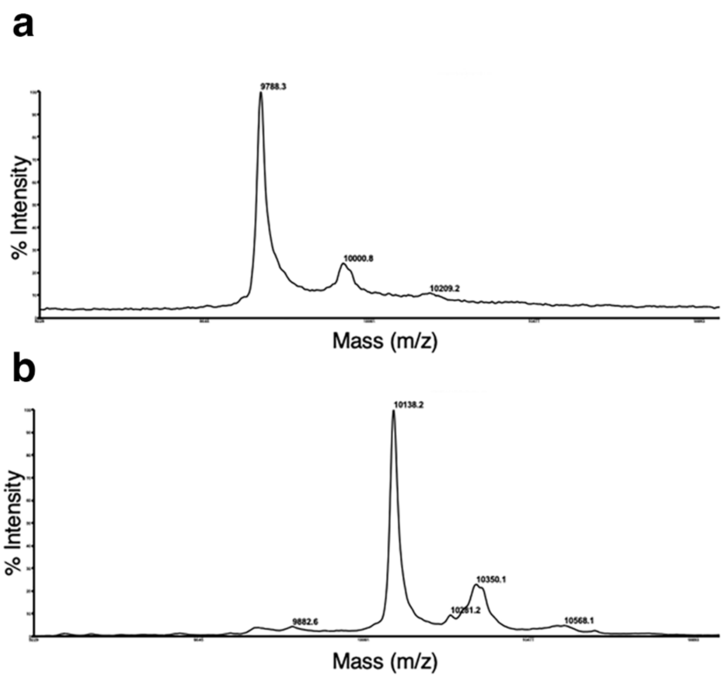

Supplementary Figure 7. Mass spectrometry analysis of apo-DltC1 (**a**) and holo-DltC1 (**b**). (**a**) MS spectra of apo-DltC1 present an ion peak with a mass-to-charge ratio of around 9788 in high relative abundance. (**b**) MS spectra of holo-DltC1 presents a peak with a mass-to-charge ratio of around 10138 in high relative abundance. The difference of 350 m/z corresponds to the phosphopantetheine group addition.

Supplementary Figure 8

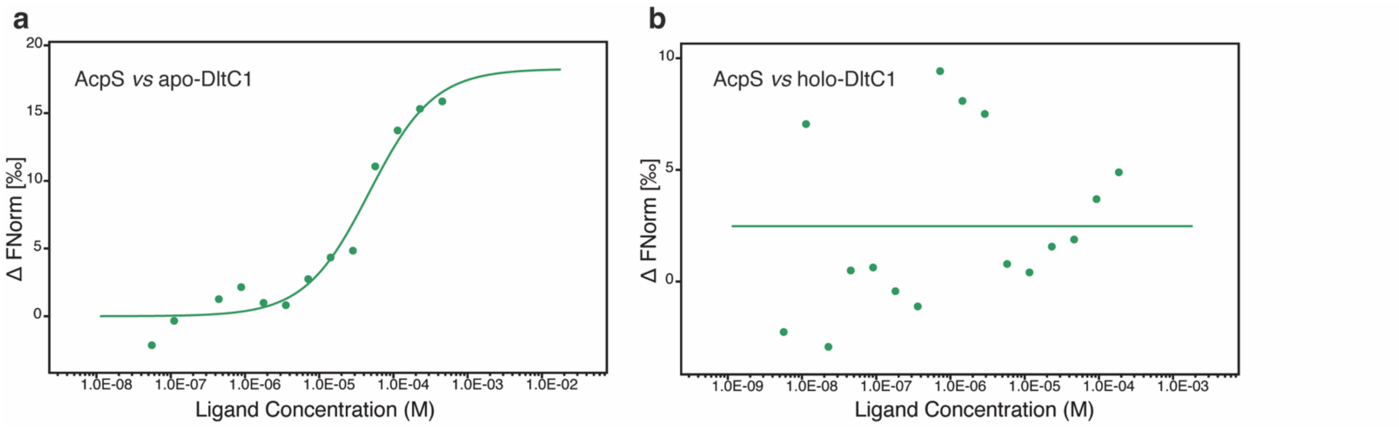

Supplementary Figure 8. Microscale thermophoresis binding assays of AcpS and DltC1. Normalized dose–response curves for the binding interaction between AcpS and apo-DltC1 (**a**) and holo-DltC1 (**b**) were obtained by plotting ΔFnorm against the ligand concentration. The data are representative of experiments made in triplicate.

Supplementary Figure 9

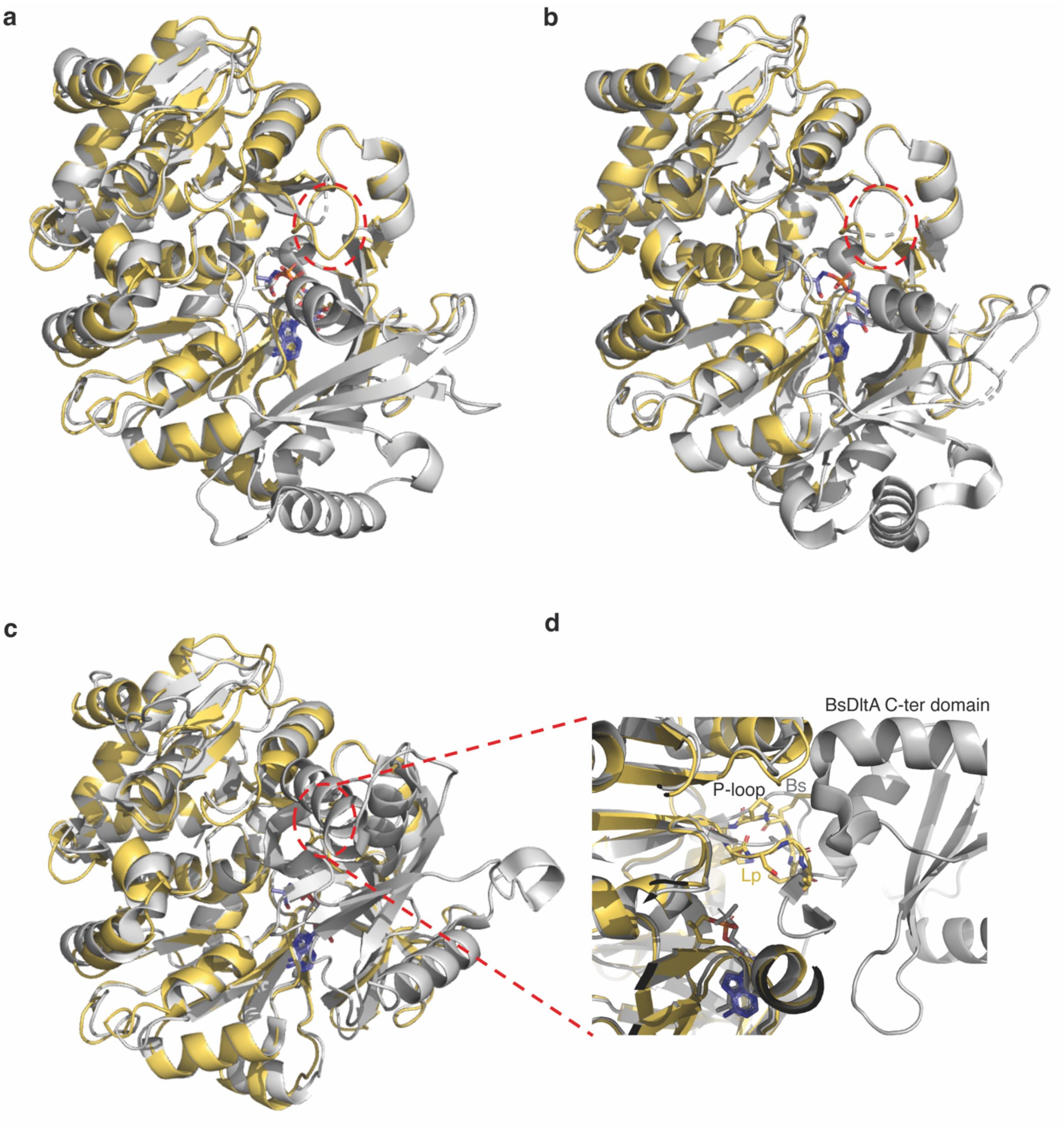

Supplementary Figure 9. Comparison of *L. plantarum* DltA structure with homologs. Superimposition of the LpDltA 3D structure depicted as yellow cartoon (this study) with in grey (**a**) the *B. cereus* DltA structure (BcDltA) in complex with D-Ala-AMP (PDB entry 3DHV) (**b**) the *S. pyogenes* DltA (SpDltA) structure (PDB entry 3L8C) in a free state and (**c**) the *B. subtilis* DltA structure (Bs DltA) in complex with AMP (PDB entry 3E7X). The P-loop from LpDltA is encircled with red dashed lines. (**d**) Close up on the P-loop from the superimposition shown in c. The side chains of the P-loop in the LpDltA structure are shown in stick.

Supplementary Figure 10

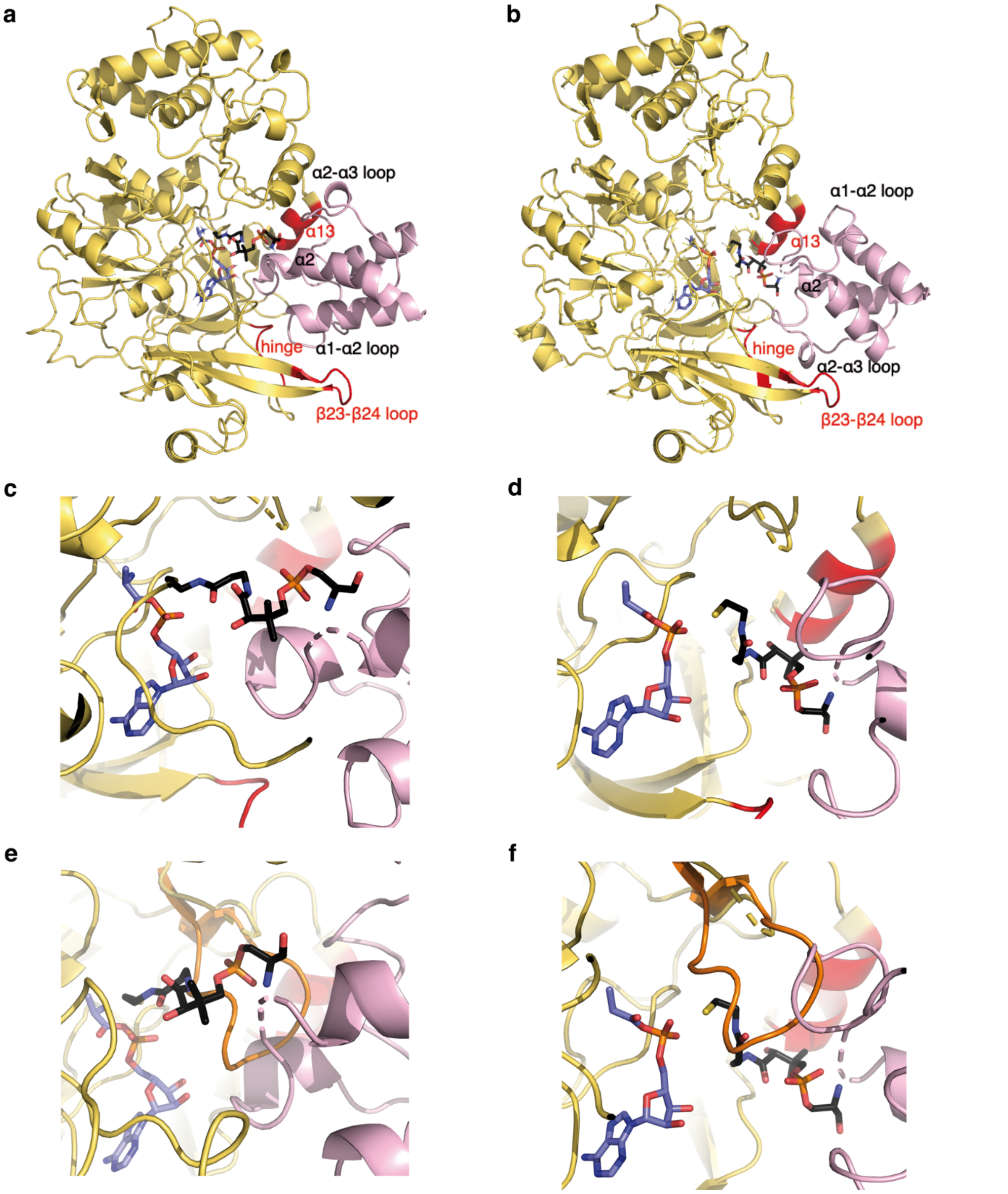

Supplementary Figure 10. Cartoon representation of the 3D model structure of the DltA/DltC1 complex (with Ppant) generated with HADDOCK. (**a** and **b**) The two best structures, 1 and 2, with the lowest intermolecular energies are shown in **a** and **b**, respectively. DltA is colored in yellow and DltC1 in pink. The predicted DltA contact regions are highlighted in red. The D-Ala-AMP molecule bound to DltA and the Ppant bound to DltC1 are shown in blue and black sticks, respectively. (**c** and **d**) Detailed view of the DltA substrate binding site. (**e** and **f**) The P-loop in the conformation observed in the *L. plantarum* DLtA X-ray structure is superimposed on the model structure of the DlTA/DltC1 complex and shown in orange.

Supplementary Figure 11

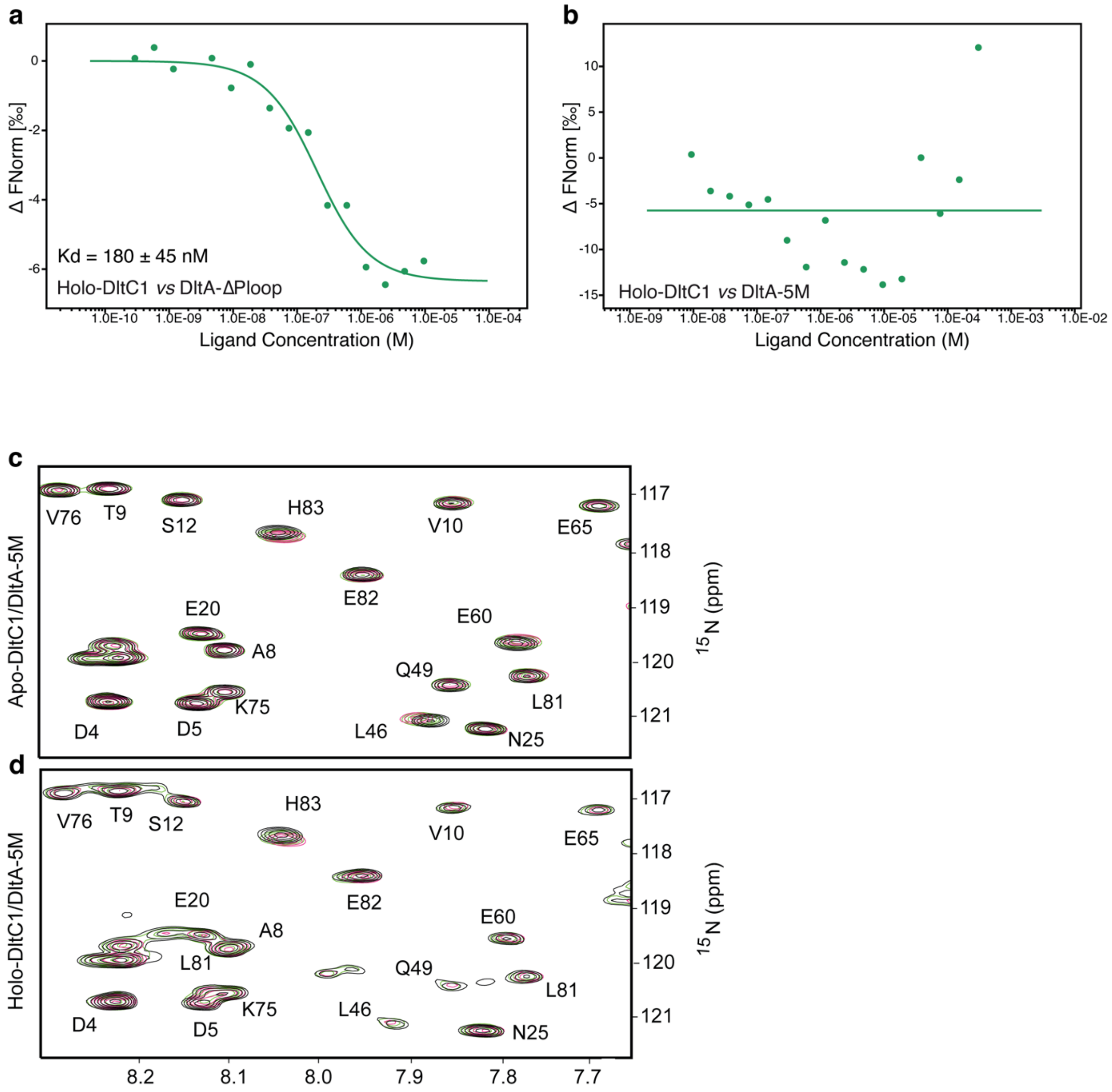

Supplementary Figure 11. Interaction between DltA mutants and DltC1**.** **(a)** and **(b)** Normalized dose–response microscale thermophoresis curves for the binding interaction between holo-DltC1 and DltA-ΔPloop **(a)** and DltA-5M **(b)** were obtained by plotting ΔFnorm against the ligand concentration. The data are representative of experiments made in triplicate. **(c)** and **(d)** 2D-[^{1}H,^{15}N]-BEST-TROSY NMR experiments of apo-DltC1 and holo-DltC1 in presence of different concentration of DltA-5M mutant. **(c)** Spectrum of ^{15}N-labelled apo-DltC1 protein prepared at 150 µM before (black) and after (green and red) addition of unlabelled DltA-5M at a ratio of 1:1.6 and 1:3.2, respectively. **(d)** spectrum of ^{15}N-labelled holo-DltC1 protein prepared 150 µM before (black) and after (green and red) addition of unlabelled DltA-5M at a ratio of 1:1.6 and 1:3.2, respectively. All the experiments were recorded at 25°C and pH 6.5.

Supplementary Figure 12

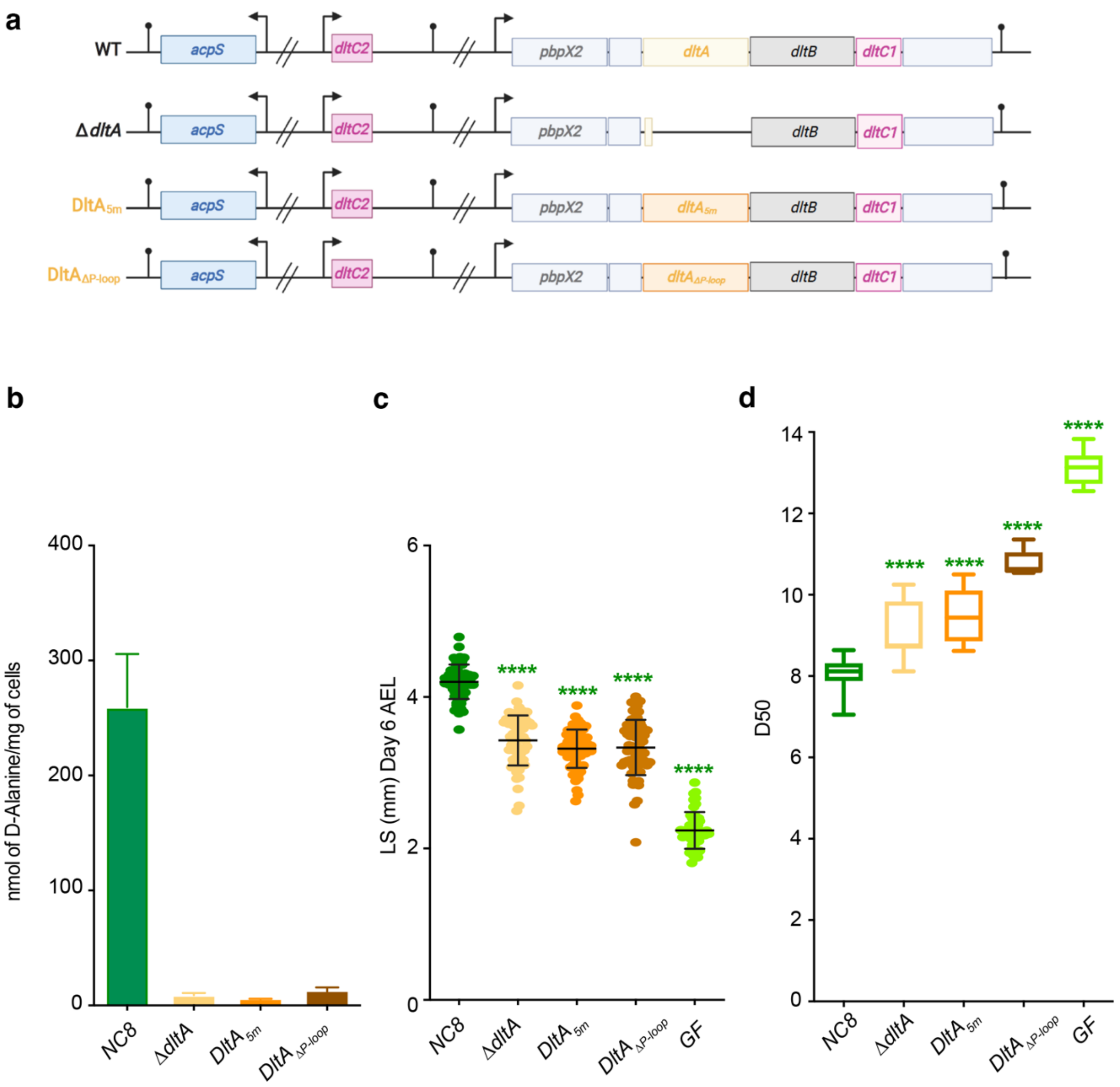

Supplementary Figure 12. Characterization of *L. plantarum dltA-5m* and *dltA*-Δ*Ploop* mutants in *Drosophila*'s growth. (**a**) Genetic organization of *Lp* engineered strains: WT corresponds to Lp^{NC8} strain; Δ*dltA* corresponds to Lp^{NC8} deleted for *dltA* gene; $DltA_{5m}$ corresponds to Lp^{NC8} harboring the *dltA* sequence with the penta-mutation; $DltA_{\Delta P\text{-loop}}$ corresponds to Lp^{NC8} strain harboring a modified version of *dltA* gene lacking the ΔP-loop region. (**b**) Amount of D-Ala released from whole cells of NC8 and derivative mutants by alkaline hydrolysis and quantified by HPLC. Error bars represent the standard deviations. (**c**) Larval longitudinal length after inoculation with strains Lp^{NC8}, Δ*dltA, dltA-5m* and *dltA*-Δ*Ploop* mutants or PBS (for the GF condition). Larvae were collected 6 days after association and measured as described in the Methods section. Green asterisks illustrate statistically significant difference with Lp^{NC8} larval size; ****: p<0,0001. Center values in the graph represent means and error bars represent SD. Representative graph from one out of three independent experiments. (d) Day when fifty percent of pupae emerge during a developmental experiment (D50) for GF eggs associated with strains Lp^{NC8}, Δ*dltA, dltA-5M* and *dltA*-Δ*Ploop* mutants or PBS (for the GF condition). Center values in the graph represent means. Green asterisks illustrate statistically significant difference with Lp^{NC8} D50; ***: 0,0001<p<0,001.

Supplementary Figure 13

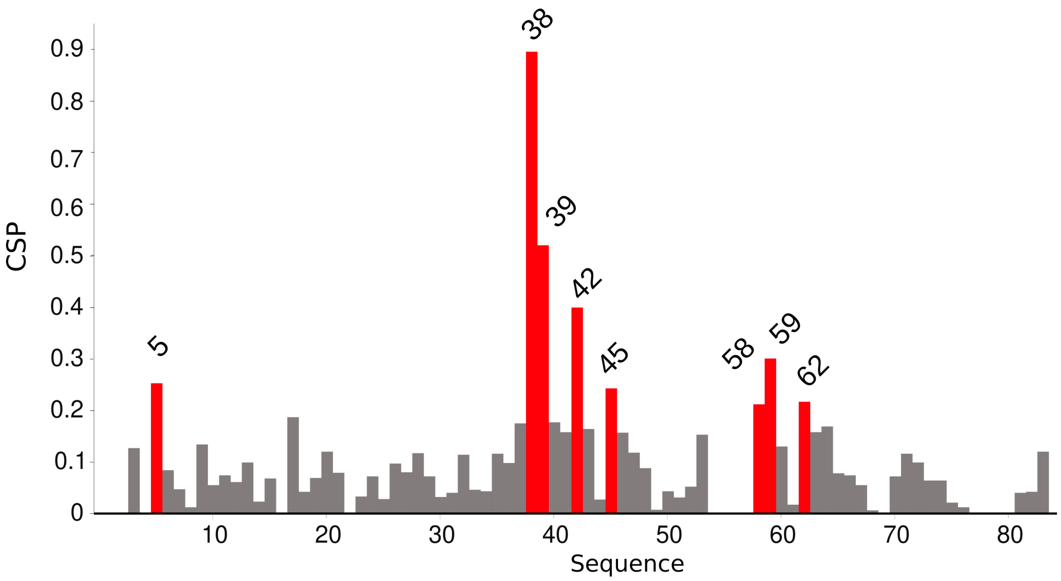

Supplementary Figure 13. Comparison of NMR spectra of apo-DltC1 and holo-DltC1. Chemical shift differences calculated from the 2D-[^{1}H,^{15}N]-BEST-TROSY NMR experiments recorded in the same conditions (25°C, pH 6. 5) on apo-DltC1 and holo-DltC1 proteins both prepared at a concentration of 150 µm. Red bars represent the value superior to 2 standard deviations calculated on all of the data (CSP > 0.2 ppm).
